# On the Origins of the Weak Folding Cooperativity of a Designed ββα Ultrafast Protein FSD-1

**DOI:** 10.1371/journal.pcbi.1000998

**Published:** 2010-11-18

**Authors:** Chun Wu, Joan-Emma Shea

**Affiliations:** 1 Department of Chemistry and Biochemistry, University of California, Santa Barbara, Santa Barbara, California, United States of America; 2 Department of Physics, University of California, Santa Barbara, Santa Barbara, California, United States of America; Centro de Investigaciones Biológicas, Spain

## Abstract

FSD-1, a designed small ultrafast folder with a ββα fold, has been actively studied in the last few years as a model system for studying protein folding mechanisms and for testing of the accuracy of computational models. The suitability of this protein to describe the folding of naturally occurring α/β proteins has recently been challenged based on the observation that the melting transition is very broad, with ill-resolved baselines. Using molecular dynamics simulations with the AMBER protein force field (ff96) coupled with the implicit solvent model (IGB = 5), we shed new light into the nature of this transition and resolve the experimental controversies. We show that the melting transition corresponds to the melting of the protein as a whole, and not solely to the helix-coil transition. The breadth of the folding transition arises from the spread in the melting temperatures (from ∼325 K to ∼302 K) of the individual transitions: formation of the hydrophobic core, β-hairpin and tertiary fold, with the helix formed earlier. Our simulations initiated from an extended chain accurately predict the native structure, provide a reasonable estimate of the transition barrier height, and explicitly demonstrate the existence of multiple pathways and multiple transition states for folding. Our exhaustive sampling enables us to assess the quality of the Amber ff96/igb5 combination and reveals that while this force field can predict the correct native fold, it nonetheless overstabilizes the α-helix portion of the protein (Tm = ∼387K) as well as the denatured structures.

## Author Summary

The protein folding process, in which a linear chain of amino acids reaches its biologically active three-dimensional shape, is fundamental to life. Small “ultrafast” folders, proteins that fold in microseconds, have received considerable attention, because these proteins serve as model systems for the folding of larger proteins, and thus permit a testing of the accuracy of computational models as well as an assessment of protein folding theories. FSD-1, a designed small ultrafast folder with a ββα fold, has been actively studied in the last few years as a model system for mixed α/β fold proteins. The suitability of this protein to describe the folding of naturally occurring proteins has however recently been challenged based on the observation that the melting transition is very broad, with ill-resolved baselines. Prior simulations have not been successful in providing an interpretation of this broad melting transition. In the present study, our extensive molecular dynamics simulations using the AMBER protein force field (ff96) coupled with the implicit solvent model (IGB = 5) shed new light on the nature of the folding transition of this protein, as well as reveal the strengths and weaknesses of the force field in predicting the thermodynamics and kinetics of folding.

## Introduction

Small “ultrafast” folders (proteins that fold on the order of microseconds), both naturally occurring and designed, have received considerable attention in the last few years. These proteins have the singular advantage of being computationally tractable, thus bridging the gap between experimental and *in silico* studies. They permit not only a testing of the accuracy of computational force fields, but also (in the event that the force field proves to be adequate) an assessment of protein folding theories. Folding mechanisms and the possibility of multiple folding pathways, both of which can be difficult to determine from standard bulk measurements, can be resolved through an analysis of molecular dynamics simulations.

FSD-1 is a 28 residue designed ultrafast folder with a ββα (hairpin/helix) fold [1]. The protein has a well-defined hydrophobic core, and unlike the more commonly studied ββα BBA5 protein (which has a D-proline at the β-turn position), only contains naturally occurring residues. The folding time of FSD-1 has not been reported, but the folding kinetics of a close analog, FSD-1ss (involving substitution of two non-natural aromatic residues at positions 6 and 26) have been monitored using laser-induced temperature-jump spectroscopy (Table 1) [2]. This modified protein displayed two folding phases (τ_1_∼150 ns and τ_2_∼4.5 µs) at 322 K, placing FSD-1ss at the top range of known ultrafast folders. Although the N-terminal region FSD-1ss (residues 16–23) adopts a loose U-shape rather than the tight β-hairpin seen in FSD-1, the overall tertiary structure of FSD-1ss is similar to FSD-1 with Cα Root Mean Square Deviation (Cα-RMSD) of only 2.2 Å. One can therefore expect that FSD-1 folds on similar microseconds timescales as FSD-1ss.

**Table 1:** Folding times for three ββα proteins FSD-1, FSD-1ss and BBA5 of similar size.

	FSD-1^a^	FSD-1ss^b^	BBA5^c^
This work	2.2±0.7 µs, 323 K	-	-
Experiments	-	4.5 µs, 322 K	7.5 µs, 298 K

a Based on the free energy barrier at 323 K.

b Ref. 2.

c Ref. 17.

The thermal unfolding of FSD-1, as determined from Circular Dichroism (CD) [1] and Differential Scanning Calorimetry (DSC) [3], is reversible, but weakly cooperative, with a relatively low melting temperature (T_m_ = 315 K). Mayo and co-workers, who designed this protein, attributed the observed transition to the melting of the entire protein. Feng et al. [3] have however recently challenged this interpretation and have proposed that the broad transition, which lacks clearly defined folding or unfolding baselines, in fact reflects only the melting of the α-helical segment (residues 14–26) of the protein. Should this interpretation be correct, then one would have to reconsider FSD-1 as a model system for studying the folding of α/β proteins.

Prior simulations of the FSD-1 protein have met with mixed (and sometimes conflicting) results, and have not provided a clear picture of the nature of the melting transition. For instance, a replica exchange molecular dynamics (REMD) simulation of FSD-1 in explicit solvent, using the Amber protein force field (ff03), TIP3P water and the NVT ensemble, predicted a melting temperature of 411.59 K [4], ∼100 K higher than the experimental value of 315 K. Simulations performed using a different force field, water model and simulation protocol (OPLS-AA/L 2001 force field, TIP4P water model and NPT ensembles with REMD simulation) lead to a melting temperature that is 84K higher than what is observed experimentally [3]. These unsatisfactory results may be the result of inadequate force fields, or/and due to insufficient sampling. In order to overcome possible sampling issues related to the use of explicit solvents, a number of groups have turned to coarse-grained protein models [5] or to implicit water models. Pak and co-workers, using the CHARMM 19 force field in conjunction with a GB solvation model [6] witnessed the folding of FSD-1 to a structure 2.56 Å Cα-RMSD from the NMR structure at 440 K in 15 ns, i.e. at a temperature well above the experimental T_m_ and with a folding time off by orders of magnitude. The authors had better success using replica exchange molecular dynamics (REMD) simulations and a newly modified version (param99MOD5) of AMBER 99 with GBSA implicit solvent [7], obtaining a computational melting temperature of ∼309 K. However, the predictive power of these simulations is uncertain given that FSD-1 was used as a training peptide in the optimization of the force field. Finally, using a newly optimized force field in combination with the recently developed implicit solvent (IGB = 5) [8], Lei and co-workers [9] were able to fold the double mutant of FSD-1 (FSDEY) [10], into its native state with high population (>64.2%) and high fidelity (1.29 Å). However, the computationally generated heat capacities failed to produce a melting transition at 315K, and the melting of the helix was observed at 360K which is higher than that of stable helical protein [11].

In the present paper, we investigate the folding of FSD-1 using the Amber ff96 protein force field combined with the implicit water solvent IGB = 5. This combination has been shown by Dill and co-workers to have a good balance of α/β propensity in the case of small peptides [12]. We have recently been able to use this combination to investigate the conformations adopted by natively disordered amyloid peptides (e.g. prion fragment [13] and amylin [14]) which can sample a variety of conformations including α, β and α/β. Recent successes of this force field/implicit solvent combination include the successful folding of the 39-residue NTL9 protein [15] by the Pande group and the unfolding of the 64-residue protein L [16]. The majority of studies involving the ff96/igb5 model have focused on assessing the model's reliability in generating accurate structural properties of proteins. Here, we present a thorough thermodynamic and kinetic analysis that enables, through a direct comparison with experimental data, an in-depth evaluation of the strength and weaknesses of this force field/implicit water model combination. Our simulations offer the first comprehensive interpretation of the broad transition at 315 K and resolve the issue of whether it corresponds to the melting of the protein or to an only helix-coil transition. Furthermore, our extensive simulations enable a thorough exploration of the energy landscape for folding, a structural characterization of transition state ensemble, and explicitly demonstrate the existence of multiple folding routes. Our simulations indicate that while ff96/igb5 can be reliably used to identify the folded state of the protein, as well as predict the thermodynamic order of formation of the structural elements in folding, there remain areas in which the protein force field/implicit water model needs to be improved. In particular, we find that ff96/igb5 overstabilizes both the denatured state and the helical portion of the protein.

## Results

### REMD simulation

#### REMD simulations predict the correct native fold

REMD simulations started from an extended chain conformation were performed as detailed in Methods section. 16 replicas ranging from 271 K to 475 K were used, each of a 1.25 µs duration, leading to a cumulative simulation time of 20.0 µs. The convergence of the REMD simulations was rigorously verified by a block analysis: the total sampling time of 1.25 µs for the replica at 280 K were equally divided into five blocks, the population of the folded structure (<3.0 Å to the NMR structure) were calculated for each block, and a good convergence was found during the 500 ns of the simulations (Text S1). All analyses were done over only the last 500 ns of the simulations.

**Figure 1:**
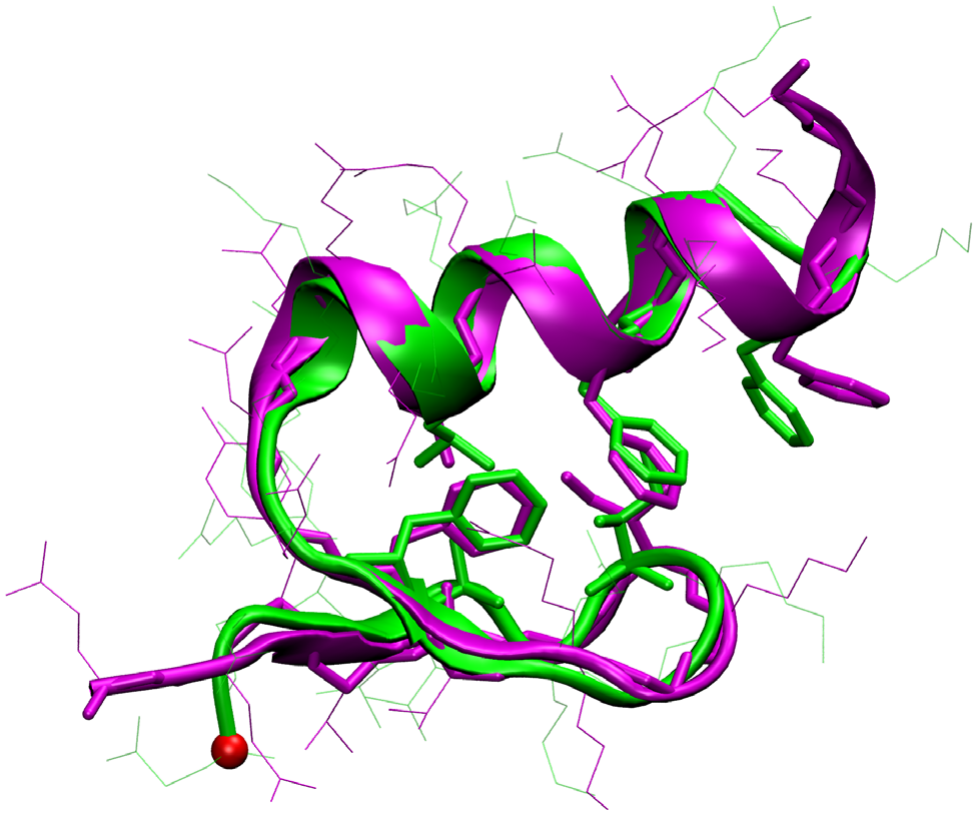
Predicted structure of FSD-1 (Green) at 280 K from REMD initiated from an extended conformation. It is 1.7 Å of Cα-RMSD compared with the NMR structure at 280 K (purple, Protein Data Bank ID code: 1FSD).

To ascertain whether our simulations can predict the correct structure of FSD-1, we follow the typical two step protocol used in the structural prediction community: 1.) identification of the most populated structure (i.e. the lowest free energy structure) by a clustering method, 2.) comparison of this structure with the experimental structure. We used a pair-wise clustering algorithm (see Methods section) to pick up the centroid structure of the most populated structural family from the ensemble at 280 K. Fig. 1 shows the comparison between our predicted structure at 280 K and the NMR structure measured at 280 K. An excellent match is found only not between the over-all backbone but also between the side chains in the hydrophobic core. The important ramification of these simulations is that the Amber 96/IGB-5 combination is capable of identifying the correct native fold as the lowest free energy structure. This force field/ implicit water combination could be very useful in structure prediction studies of proteins whose folds have not yet been experimentally characterized.

#### Thermodynamics of folding

**Figure 2:**
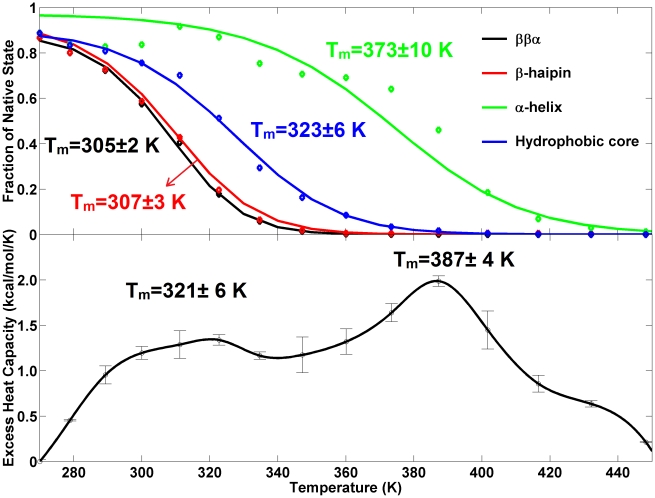
Thermodynamic features obtained from REMD simulations. Top: The fitted melting curves of different structural elements. The original data points are indicated by diamonds and the obtained melting temperatures are noted. Bottom: The excess heat capacity as a function of temperature. Standard error is obtained by dividing data into two blocks for analysis.

The melting transition can be objectively analyzed from the heat capacity profile. The sharp slope at the lowest temperatures makes it difficult to determine the native state baseline necessary for getting the excess heat capacity profile. In our analysis, we set the absolute heat capacity value at the lowest temperature as the native state baseline. The resulting excess heat capacity profile (Fig. 2, bottom) has two broad peaks at temperature 321±6 K and 387±4K indicative of two distinct melting transitions. The denaturation enthalpies for the two transitions are 70.0 kcal/mol and 122.0 kcal/mol, respectively.

The structural nature of the two transitions can be identified from the van't Hoff analysis (see Methods section) on the melting of the various structural components of FSD-1 (the C-terminal α-helix, N-terminal β-hairpin, the hydrophobic core and the whole protein). The upper panel of Fig. 2 shows the fitted curves and the thermodynamic parameters are listed in Table 2. The curve fitting is good for all structural components except for the α-helix. The lack of sharpness of the transitions in all curves indicates a weak cooperativity. The melting temperatures are 305±2 K, 307±3 K, 323±6 K and 373±10 K for the tertiary fold, the N-terminal β-hairpin, the hydrophobic core and the C-terminal α-helix. Given the correspondence between the transitions temperatures from the excess heat capacity profile and the melting curves, we conclude that: (1) the first transition at lowest temperatures (321±6 K) corresponds to the formation of the hydrophobic core (323±6 K), the N-terminal β-hairpin (307±3 K) and the tertiary fold (305±2 K). (2) the spread (∼323 K to ∼305 K) in the melting temperatures of the hydrophobic core, β-hairpin and tertiary fold accounts for the broadness of the first transition; (3) the second transition at higher temperatures (387±4K) involves the formation of the α-helix (373±10 K). Since the melting temperature of even a very stable helix is less than 343 K [11], the melting temperature of the second transition appears to be overestimated, likely a result of an imbalance in the ff96/igb5 force field parameters. Nonetheless, given that the experimental CD spectrum at elevated temperatures (353 K) shows the presence of helical structure [3] in addition to random coils, it appears that the helix is more stable than the other structural elements (although certainly not to the extent seen in our simulations). Hence the relative stability order of the four structural components seen in our simulations should still be correct (i.e. the helix is still slightly more stable than the hydrophobic core and the helix-to-coil is the last step of the denaturation). In other word, the order of the melting temperatures for the four components (373±10 K>323±6 K>307±3 K>305±2 K) suggests the following thermodynamic folding sequence: Initial formation of the C-terminal α-helix and followed by the hydrophobic core and thereafter the simultaneous formation of the N-terminal β-hairpin and the tertiary fold. (Again, since the force field likely overstabilizes the α-helix, the initial formation of the C-terminal α-helix and the hydrophobic core should be more concurrent than is seen in simulation, with a more realistic temperature gap being much smaller (≪50 K) than seen here. In terms of the van't Hoff thermodynamic parameters (i.e. the changes of enthalpy 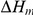, and heat capacity 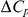 at 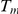) for each structural component (see Table 2), the values are generally in good agreement with experiment (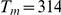
K, 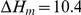 kcal/mol, 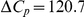
cal/mol/K). The agreement is less satisfactory with regard to the melting temperature of the α-helix (373±10 K), consistent with our earlier observations, and the heat capacity changes for the tertiary fold, (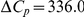
cal/mol/K) and for the N-terminal β-hairpin (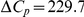
cal/mol/K). Overall, the qualitative agreement between the simulations and the CD experiment supports the interpretation that the melting of these four structural components is involved in the denaturation transition.

**Table 2:** Calculated and experimental thermodynamic parameters.

Measurement	Cutoff (Å)	T_m_ (K)	ΔH_m_ (kcal/mol)	ΔC_p_ (cal/mol/K)
CD experiment^a^	-	315	10.4	120.7
melting of ββα^b^	Cα-RMSD = 3.0	305±2	14.4±3.7	336.0±78.0
melting of β-hairpin^b^	Cα-RMSD = 3.0	307±3	13.6±3.0	229.7±66.0
melting of hydrophobic core^b^	Rg = 7.7	323±6	11.6±3.2	188.0±60.0
melting of α-helix^b^	Cα-RMSD = 3.7	373±10	13.6±4.0	125.1±50.0
DSC experiment^c^	-	314	12 to 15	-
excess heat capacity peak 1^b^	-	321±6	70.0	-
excess heat capacity peak 2^b^	-	387±4	122.0	-

a Ref. 7.

b This work (see Fig. 2).

c Ref. 3.

Despite the qualitative agreement with the experimental CD data, comparison to experimental thermodynamic data shows a number of differences with our simulations that point to some deficiencies in the simulation protocol or/and the force field/implicit water model used. The primary discrepancy lies in the fact that we see two peaks in our theoretical heat capacity plots, while the DSC experiment [3] only shows one broad peak at a temperature 314 K with a denaturation enthalpy of 12–15 kcal/mol (Table 2). The experimental peak corresponds well with the first peak in our simulations in terms of location. The presence of a second high temperature peak (not seen in experiment) can be attributed to: 1) the high temperatures (up to 465K) used in the REMD simulations (in contrast, the temperature range in the DSC experiment was 283 K–353 K); 2) the force field/implicit water model related overstabilization of the α-helix which would lead to an artificially high melting temperature for this structural element. Tuning of the force field to better balance the relative stabilities of the helical and sheet components might shift the second peak down in temperature so as to overlap with the first peak. The second difference to account for is the elevated denaturation enthalpy in the first peak (70.0 kcal/mol. vs. the experimental value of 14 kcal/mol). This could be due once again to deficiencies in the ff96/igb5 model, or to the fact that we used a Langevin thermostat with a low friction coefficient. To examine the latter, we performed the same REMD simulations, but this time with a Berendsen thermostat with a coupling constant of 2.0 ps (unpublished data). In this case, we obtain a similar heat capacity profile with two peaks (Text S1), but the native state baseline is well resolved and the denaturation enthalpy of the first peak is now 12.8 kcal/mol, in good agreement with experiment. Hence the thermostat appears to be the primary reason for the difficulty in defining the native state baseline and the overestimation of the denaturation enthalpy for the first peak. A detailed comparison between different thermostats will be reported in future work.

#### Folding free energy plot and transition state

**Figure 3:**
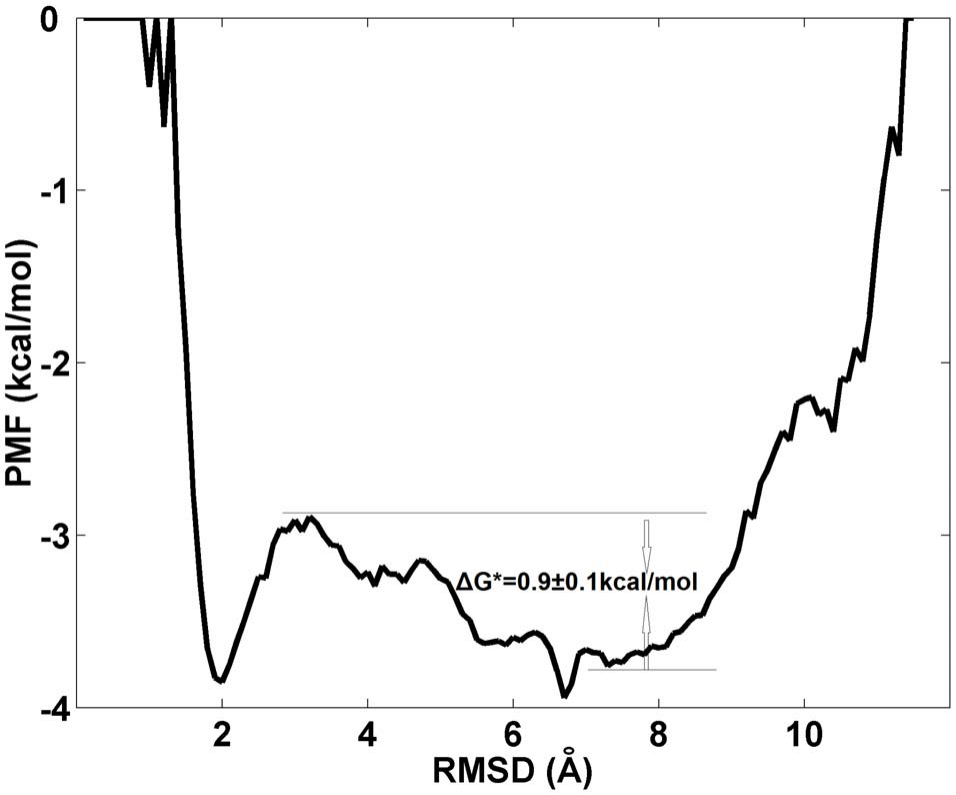
Folding free-energy of FSD-1 against Cα-RMSD of the whole protein at 323K.

**Figure 4:**
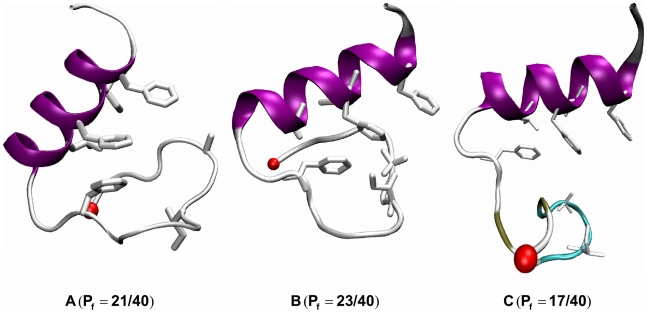
Transition structures identified from MD simulations. A: Identified from the 323 K replica of the REMD simulation by clustering analysis. B–C: Identified from the two successful CMD folding trajectories at 300 K. *P*_fold_ test results for TS states are noted.

The folding free energy surface at 323K (close to the melting temperature of 321±6 K identified form the heat capacity profile) is plotted as a function of the Cα-RMSD of FSD-1 in Fig. 3. The plot shows two basins, a narrow one centered at Cα-RMSD = 2.0 Å and a broader one centered at Cα-RMSD = 7.0 Å, separated by a barrier at Cα-RMSD = 3.0 Å. The first basin represents the folded state, whereas the second represents the unfolded state. A common feature of the structures residing at the barrier is full formation of the C-terminal helix and partial formation of the hydrophobic core and the N-terminal β-hairpin. If the order parameter chosen (Cα-RMSD) is close to the true reaction coordinate, then the barrier region should contain transition state structures. We identify a representative/centroid structure (Fig. 4A) from our clustering of the conformations present in the barrier region and use it for a *P*_fold_ test. In the *P*_fold_ test, 40 constant temperature molecular dynamics simulations (200 ns of each) are initiated from this structure and the number of folding and unfolding trajectories are counted. For this structure, about half of trajectories folded and other half unfolded (*P*_fold_∼0.5), intimating that this structure belongs to the transition state ensemble and that the Cα-RMSD might be a satisfactory reaction coordinate. (While we have identified a transition state structure from this analysis, it is difficult to comment on “how good” a reaction coordinate the Cα-RMSD really is. A “true” reaction coordinate would contain *only* transition state structures at the barrier region, this constituting the necessary and sufficient condition for being a reaction coordinate. An analysis of all the structures residing at the top of the barrier to assess the quality of the reaction coordinate would simply be computationally prohibitive). Next, we use the barrier height to estimate the folding time. Following Kramer's theory (see the method section for details), the folding time is estimated to be τ_folding_ = 2.2±0.7 µs from equation 7 by input of the free energy barrier (ΔG* = 0.9±0.1 kcal/mol) and pre-exponential factor (*A* = 0.56 µs). The order of magnitude is comparable to those of two similar proteins [2], [17] (e.g. similar size and the same fold) listed in Table 1. In particular, this folding time is of the same order as the one (4.2 µs) measured at 322 K for the fluorophore mutant (FSD-1ss) of FSD-1. (We note that because our reaction coordinate is not the “true” reaction coordinate, our computational value is a lower bound estimate of the true folding time).

### Conventional Molecular Dynamics (CMD) simulations

**Figure 5:**
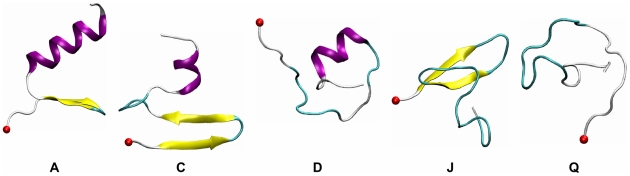
Representative structures from the last snapshots of 20 CMD trajectories. The backbone is shown in cartoon and the secondary structure is coded by color: coil in silver, α-helix in purple, β-sheet in yellow, isolated β-bridge in tan and turn in cyan.

To directly probe folding routes, we performed 20 CMD simulations (1 µs each) at 300K starting from an extended conformation. The last snapshot of each trajectory (Text S1) offers a quick structural assessment: a successful α/β fold was formed in 2 trajectories (A–B), an unpacked α/β fold was observed in 1 trajectory (C), a partial α-helix was formed in 6 trajectories (D–I), a partial β-sheet was formed in 7 trajectories (J–P) and a compact coil-turn fold was formed in 4 trajectories (Q–T). A representative structure for each structural family is shown in Fig. 5. Altogether, 10% of the trajectories folded to the correct α/β fold, yielding an estimated folding time of 10±7 µs (see Methods section). This time needs to be corrected taking into account the fact that our simulations used a friction coefficient 1/60 of the one in water. The corrected folding time is at least 600 µs, far larger than our estimate of 2.2 µs from the barrier height from REMD (or the 4.5 µs folding time of the FSD1-ss mutant). The implication is that the CMD simulations lead to a much rugged folding landscape than “reality” and that the ff96/igb5 combination may overstablize unfolded structures (e.g. C–Q in Fig. 5).

**Figure 6:**
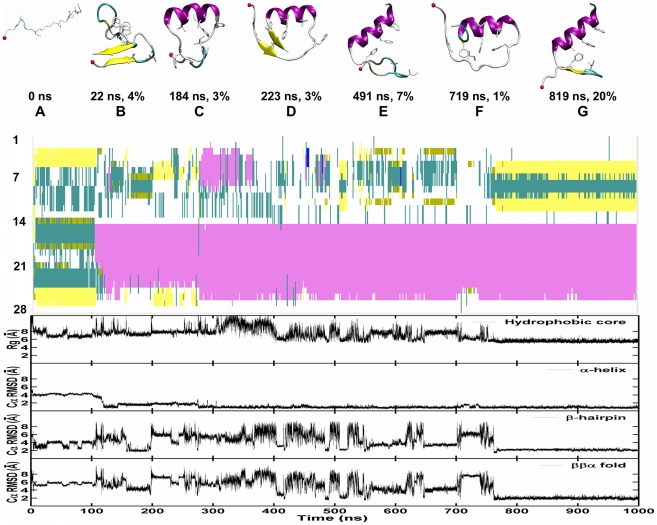
Successful folding trajectory 1 from CMD simulations of FSD-1 at 300 K. Top: The representative snapshots of top structural families are presented together with their abundance and time. The backbone is shown in cartoon. The secondary structure is coded by color: coil in silver, α-helix in purple, β-sheet in yellow, isolated β-bridge in tan and turn in cyan. N-terminal is shown by a red VDW ball. Middle: The development of secondary structure. Bottom: The development of four order parameters: the radius of gyration of the hydrophobic core, the Cα-RMSD of the N-terminal hairpin (residues 3–13), the C-terminal α-helix (14–26) and the whole protein (residues 3–26) against the NMR structure.

**Figure 7:**
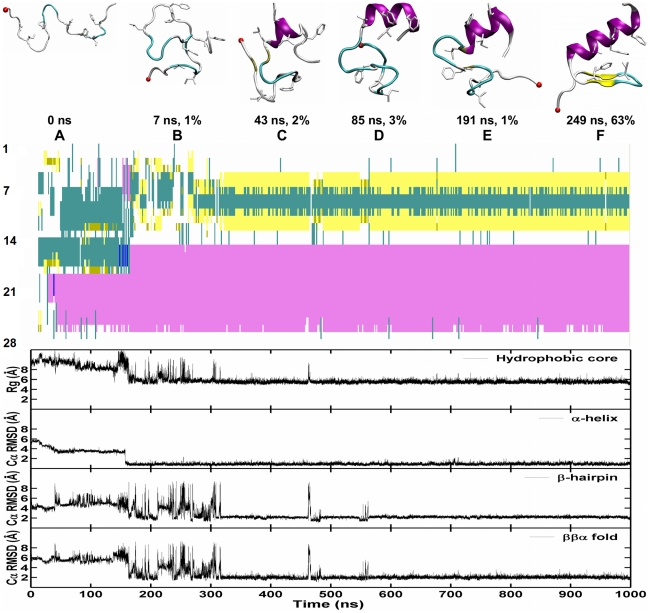
Successful folding trajectory 2 from CMD simulations of FSD-1 at 300 K. Top: The representative snapshot of top structural families is presented together with its abundance and time. The backbone is shown in cartoon. The secondary structure is coded by color: coil in silver, α-helix in purple, β-sheet in yellow, isolated β-bridge in tan and turn in cyan. N-terminal is shown by a red VDW ball. Middle: The development of secondary structure. Bottom: The development of four order parameters: the radius of gyration of the hydrophobic core, the Cα-RMSD of the N-terminal hairpin (residues 3–13), the C-terminal α-helix (14–26) and the whole protein (residues 3–26) against the NMR structure.

The Cα-RMSD of each trajectory (Text S1) was calculated (defined as Cα-RMSD<3.0 Å for at least consecutive 30 ns) for each trajectory. The two successful folding trajectories are shown in Fig. 6 and Fig. 7. By applying the clustering analysis described in the Methods section to each trajectory, we identified structural families whose population is greater than 1% of the total snapshots. The representative structures (the centroid of each structural family), with their respective time of occurrence and abundance, are shown in Fig. 6 and Fig. 7. Strikingly, two different folding routes are observed in these two trajectories.

#### Trajectory 1

Following hydrophobic collapse, a non-native main chain fold (A) emerges, consisting of a short two-strand antiparallel β-sheet formed at the two ends, a large disordered loop and a non-native hydrophobic core. The non-native main chain fold and the non-native hydrophobic core then unfold and the correct C-terminal native α-helix (B) forms, initiating from the middle of the protein chain and rapidly extending to the C-terminus (within 20 ns). The N-terminal fragment sampled various conformations (non-native ones in structures D and F, and native-like in structures C and E) before reaching the native β-hairpin (G). Some features are noted from the time development of the 4 order parameters: 1) the formation of C-terminal α-helix (within 120 ns) preceded the formation of the N-terminal β-hairpin, hydrophobic core and global ββα fold (within 780 ns); 2) the formation of N-terminal β-hairpin was highly correlated to the formation of the tertiary fold with a correlation coefficient of 0.88, indicating a concurrent assembly of the β-hairpin and tertiary fold; 3) the formation of the hydrophobic core was moderately correlated with the folding of the tertiary fold with correlation coefficients of 0.69, suggesting a spread between the formation of the hydrophobic core and the tertiary fold.

#### Trajectory 2

After collapse, a compact disordered structure without any hydrophobic core (B) was formed. The C-terminal native α-helix (B) was initiated from the lower half of the protein chain and extended toward both the C-terminus and the N-terminus (within 140 ns). Again, the N-terminal fragment sampled various conformations (the native-like one in C, and non-native ones in D and E) before reaching the native β-hairpin (G). Trajectory 2 shares the main three features observed in Trajectory 1 (and listed in the previous paragraphs), yet the details of the folding pathway differ, and folding occurs faster (320 ns).

In summary, both trajectories share the following common features: the early formation of the α-helix within 150 ns, and the concurrent formation of β-hairpin and tertiary structure within 320–780 ns. These features are consistent with the thermodynamic data obtained from the replica exchange simulations. Despite the overall similarity in folding mechanism, the individual trajectories show clear differences in the details of how the individual components form: 1) the C-terminal helix can initiate from different sites (residues 15–20 in Trajectory 1 vs. residues 19–22 in Trajectory 2); 2.) A significant heterogeneity exists across the two successful folding trajectories (for instance, trajectory 1 shows early transient population of a structure with two β-strands). Our direct observation of multiple folding routes is in direct line with the folding funnel energy landscape perspective [18] .

#### Transition state structures from the two folding trajectories

Putative TSS were identified from both trajectories as the structures that will fold into the native structure in the next time step [19]. 40 independent simulations starting from the putative structures (200 ns of each) were performed to calculate a *P*_fold_ value and determine whether or not these conformations belong to the transition state ensemble. For the two structures shown in Fig. 4 (B–C), (each coming from one of the two folding trajectories), we observed about a half of 40 trajectories (23 and 17) reaching the native state. Clearly, these two “kinetic” transition state structures share similar structural features to the “thermodynamic” transition state structures obtained from the REMD data (Fig. 4A): full formation of the C-terminal helix and partial formation of the hydrophobic core and the N-terminal β-hairpin. The major difference between these structures, which reflects the heterogeneity of the transition state ensemble, is located at the β-hairpin part of FSD-1.

## Discussion

FSD-1, a designed small ββα ultrafast folder, has received considerable experimental and computational attention as a model system for studying protein folding mechanisms and for testing computational models. However, the validity of using FSD-1 as a prototypical folder has recently been challenged by Feng et al [3]. They argue that the broad melting transition at 315 K observed by CD and DSC is mainly due to the melting of the helical portion of the protein, rather than to the melting of the entire protein. They point out that an overall protein transition should exhibit a better-defined baseline and a higher melting temperature (for comparison, HP35 folds at 342 K) and the β-hairpin part appears to be flexible and lack of stability based on their REMD simulations using OPLS-AA/L 2001 force field, TIP4P water model and NPT ensemble. Their simulations and others [4], thus far, have not been able to address this controversy and provide an explanation for the broad melting transition of FSD-1, with computational folding transitions all lying at much higher temperatures than the experimental ones. Reliable folding of mixed α/β fold proteins like FSD-1 is notoriously difficult, mostly because most force fields are either heliophilic [20], [21] or β-centric [12].

In the present paper, we investigate the folding of FSD-1 using the Amber ff96 force field combined with the implicit solvent IGB = 5, which appears to offer a reasonable balance of helical and beta propensities. Our REMD simulations show two broad peaks in the excess heat capacity at T_m_ = 321±6 K and T_m_ = 387±4K. These peaks correspond to the two structural transitions identified from the computational thermal-denaturing curves: 1) formation of the hydrophobic core (T_m_ = 323±6 K), β-hairpin (T_m_ = 307±3 K) and tertiary fold (T_m_ = 305±2 K) and 2) formation of the α-helix (T_m_ = 373±10K). By comparing the simulations results with the DSC and CD experiments, we find that the first transition qualitatively agrees with the experiments, whereas the second transition is an artifact of the simulations resulting from the ff96/igb5 induced overstabilization of the α-helix. This suggests that the improved igb5 solvation model does not fully resolve the secondary balance problem inherent to the heliophilic Amber ff96 force field and that further refinement of the protein force field is necessary.

While the force field clearly overstabilizes the helix, the fact that the helix is more stable than the other secondary structural elements is confirmed by the CD spectra. A re-examination of the experimental CD spectra of FSD-1 at 353K and 277 K presented in Fig. 2 of ref 3 shows that the spectrum above the experimental T_m_ = 315 K possesses helical features rather than coil-only features and the CD spectrum below the experimental T_m_ = 315 K possesses both β-hairpin-rich and helix-rich features rather than helix-only features. Indeed, the spectrum at 353 K lacks random-coil features (i.e. very low ellipticity above 210 nm and negative bands near 195 nm); instead it shows helical negative bands at 203 nm and 222 nm, indicating the presence of helical structure. In addition, the CD spectrum of FSD-1 at 277 K not only shows two helix bands at 207 and 220 nm but also contains a sheet band at 218 nm, intimating that the protein shows signs of a folded α/β protein at this temperature. Furthermore, the CD spectrum of FSD-1 is significantly different form the CD spectra of the helix-only protein [11], suggesting it contains both α and β secondary structure.

Put together, our prediction is as follows: although the α-helix is the most stable structural component (i.e. the helix-to-coil transition is dominant in the denaturation process), the broad denaturation transition seen experimentally also has a significant contribution arising from the spread in melting temperatures of the hydrophobic core, the β-hairpin. In other words, the breadth does not arise solely from the helix-coil transition as had been proposed by Feng and coworkers [3]. Our simulations explain the experimentally observed lack of well-defined baselines as due to the marginal stability of this protein, coupled with the spread in melting temperatures of the individual secondary and tertiary components. We suggest that mutations that would enhance the stability of the β-hairpin could improve the overall stability and the cooperativity of this designed protein, making it an even better model for natural α/β proteins.

The folding free energy landscape at temperatures near the folding temperature (Tm = 321±6 K) shows two well-defined folded and unfolded basins. Transition state structures identified from the structures at the top of the barrier satisfying the *P*_fold_ analysis revealed common features: full formation of the C-terminal helix and partial formation of the hydrophobic core and the N-terminal β-hairpin. The free energy barrier height enables us to calculate the folding time of FSD-1. Following Kramer's theory, the folding time is estimated to be τ_folding_ = 2.2±0.7 µs, which is of the same order of magnitude as that of FSD-1ss [2], which shows two folding phases (τ1∼150 ns and τ2∼4.5 µs). Should FSD-1 present the same two phases, we could assign the first fast phase to the formation of hydrophobic core, and assign the concurrent formation of N-terminal β-hairpin and the tertiary fold to the slower phase (∼4.5 µs).

Furthermore, our CMD trajectories provide detailed atomic information of possible folding routes starting from a straight chain. Although the general folding features gleaned from the REMD thermodynamic analysis (e.g. early formation of the C-terminal helix and thereafter folding N-terminal hairpin and the tertiary fold) are seen in the two successful folding trajectories, a significant heterogeneity is present across the two folding trajectories: 1) the C-terminal helix can initiate from different sites (residues 15–20 in Trajectory 1 vs. residues 19–22 in Trajectory 2; 2) different structures were visited along the folding routes; 3) the transition state structures found in each trajectory, validated by *P*_fold_ analysis, differ, supporting the notion of different transition structures for multiple routes [22]; 4) significant non-native topologies and secondary structures are also sampled along the two folding routes. The presence of these structures underlines the importance of considering non-native interactions, in addition to the natively favored interactions used in Go-like models [23]. Non-native conformations play a role in modulating folding times and mechanisms. Put together, our all-atom CMD data provide direct evidences to a funneled energy landscape [18] with multiple folding pathways and a diverse transition state ensemble. Nonetheless, the low number of successful folding runs (only 2 out of 20 trajectories) and the resulting lengthy folding time (∼600 µs vs. 2.2 µs) indicates that the CMD simulations generate a much more rugged folding landscape than reality. A better modeling by the implicit protein force field for the unfolded state might resolve the problem.

It is interesting to compare the folding mechanism of FSD-1 to a similar protein (BBA5) with ββα fold. In the case of BBA5, Pande and coworkers showed that this protein follows a diffusion-collision model [17], [24]: docking of the preformed α-helix and β-hairpin. Thus, for BBA5, the formation of the β-hairpin precedes the formation of the tertiary fold, whereas in the case of FSD-1, we have shown that the β-hairpin and the tertiary fold form concurrently. Clearly, the D-proline present at the turn region of the β-hairpin of BBA facilitates the formation of this structural element, but, as a result, it may reduce the overall folding cooperativity of the protein. It is possible that the introduction of non-natural amino-acids makes this protein less “funnel-like”. Nonetheless, BBA5 and FSD-1 fold in microsecond despite these differences in folding mechanism, consistent with contact order theory that suggests that native topology is a major factor in determining the folding time [25].

## Methods

The AMBER 8 simulation package [26] is used in both molecular dynamics simulations and data processing. The protein is modeled using the AMBER all-atom point-charge force field, ff96 [27]. Solvation effects are represented by the recent implicit solvent model (IGB = 5) [8] plus the surface term (gbsa = 1, 0.005 kcal/Å^2^/mol) with an effective salt concentration of 0.2 M.

### REMD and CMD folding simulations started from an extended chain conformation

An initial energy minimization was performed on an extended chain conformation and the minimized structure was used as the input for both replica exchange molecular dynamics (REMD) and conventional molecular dynamics simulations (CMD) simulations. In REMD simulation [28], [29], [30], [31], 16 replicas were set up with initial temperatures exponentially spaced from 271 to 465 K for solution phase calculations (i.e 271.0 280.0 289.3 300.0 311.2 322.7 334.7 347.2 360.1 373.4 387.3 401.7 416.6 432.1 448.2 465.0, see reference [32] for the algorithm used to optimize them). 20 CMD simulations were conducted at 300 K. Initial velocities for each trajectory were generated according to the Maxwell-Boltzmann distribution for its target temperature. The first 1.0 ns of REMD simulation was performed to equilibrate the system at its target temperatures. After equilibrium, exchanges between neighboring replicas were attempted every 1000 MD steps (2.0 ps) and the exchange rate among replicas was ∼20%. SHAKE [33] was applied to constrain all bonds connecting hydrogen atoms and thus a time step of 2.0 fs. In order to reduce computation time, non-bonded forces were calculated using a two-stage RESPA (reference system propagator algorithm approach) [34] where the forces within a 12 Å radius were updated every step and those beyond 12 Å were updated every two steps. The Langevin dynamics was used to control the temperature 300K using a collision frequency of 1.0 ps^−1^. The lower collision frequency than a typical value (∼60 ps^−1^) for water solvent was used for a better conformational sampling. The center of mass translation and rotation were removed every 250 MD steps (0.5 ps). Each trajectory was run for 1.25 µs and 1.0 µs respectively in REMD and CMD simulations, giving a cumulative simulation time of 20.0 µs. The trajectories were saved at 2.0 ps intervals for further analysis.

### Secondary structure and tertiary structure analysis

For analysis of secondary structure, the STRIDE program of Frishman and Argos [35] is used. For analysis of tertiary structural families, the snapshots are clustered by the GROMACS protocol [36], in which the structure similarity score is based on pair wise Cα-RMSD of 2.0 Å. This is done in order to reduce a large number of the sampled structures into a few structural families. The structure with the largest number of neighboring structures within the cutoff, was selected as the representative structure of the structural family/cluster.

### Four order parameters

The formation of hydrophobic core, secondary structures and tertiary structure are important events in the protein folding process. To monitor the formation of the hydrophobic core of FSD-1, we calculate the radius of gyration for the hydrophobic core formed by the residues Ala5, Ile7, Phe12, Leu18, Phe21, Ile22, and Phe25. To characterize the secondary and tertiary structure formation, the Cα-RMSDs of the N-terminal hairpin (residues 3–13), the C-terminal α-helix (residues 14–26) and the whole protein (residues 3–26), are calculated against the NMR structure (pdb id: 1FSD). The terminal residues are flexible and thus are not included the calculation.

### Thermodynamic properties

The fractions of the folded state 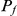 at various temperatures are directly calculated from the REMD trajectories based on each of the four order parameters. Here, the folded state is defined by setting a cutoff of Cα-RMSD or the radius of gyration (Rg) for each order parameter. The cutoff is the value that separates the two populations in the distribution of each order parameter at 323 K (Text S1). The values are listed in Table 2.

The melting transition temperature 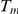 is obtained at the midpoint where 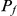 = 0.5. Assuming a two-state thermodynamic model, the thermodynamic parameters in the direction of folding to unfolding can be obtained from the following van't Hoff analysis [37]:
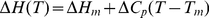
(1)

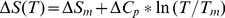
(2)

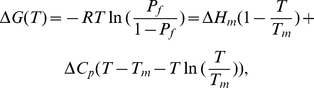
(3)where 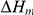, 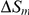 and 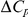 (assumed to be constant across temperature) are the changes in the van't Hoff enthalpy, entropy at 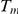 and heat capacity at constant pressure.

Also, 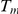 (i.e. peak temperature values) and the denaturation enthalpy,
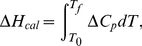
(4)can be obtained from the excess heat capacity profile, which is calculated by subtracting the heat capacity of the native state (i.e. the lowest temperature) from the absolute heat capacity profile [38]:
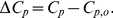
(5)The absolute heat capacity (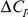) is estimated from the potential energy distribution at each temperature from the REMD simulation by
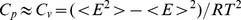
(6)where *E* is the potential energy, *R* is the gas constant, and T is the temperature.

**Estimation of the folding time:** The folding time 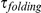 can be obtained from Kramers' theory of unimolecular reaction rates in solution [39], [40], [41]:
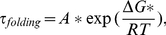
(7)where 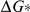 is the height of the free energy barrier, *R* is the gas constant and *T* is the absolute temperature, *A* is the pre-exponential factor. As to the 40-residue protein BBL, *A* is about 0.8 µs [42] estimated by the measured relaxation time [43] of the Förster resonance energy transfer (FRET) efficiency for the acid-denatured state of the BBL with donor and acceptor fluorophores attached to the N and C termini at 305 K. Using the linear length scaling suggested by the homopolymer collapse theory [44], the pre-exponent factor *A* for a 28 residue FSD-1 is about 0.56 µs (0.80 µs * 28/40).

Alternatively, the folding time 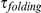 based on two-state folding model can be estimated from a large number of CMD simulations (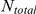) of a short duration (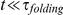) [45]:
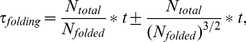
(8)where 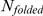 is the number of the folded trajectories.

## Supporting Information

Text S1 Analysis of folding simulation data. Block analysis of the REMD, distribution of the four order parameters at 323K, heat capacity profile using Berendsen thermostat, RMSDs of 40 CMD trajectories from TS1, Final snapshots and RMSDs of 20 CMD trajectories from an extended conformation and RMSDs of 80 CMD trajectories from TS2 and TS3 are included.
(4.42 MB DOC)
