# Supplementary material for: On the Origins of the Weak Folding Cooperativity of a Designed ββα Ultrafast Protein FSD-1
Source: PLoS Comput Biol. 2010 Nov 18;6(11):e1000998. doi: 10.1371/journal.pcbi.1000998 (PMC2987907; doi:10.1371/journal.pcbi.1000998)
Supplement: Text S1 — Analysis of folding simulation data. Block analysis of the REMD, distribution of the four order parameters at 323K, heat capacity profile using Berendsen thermostat, RMSDs of 40 CMD trajectories from TS1, Final snapshots and RMSDs of 20 CMD trajectories from an extended conformation and RMSDs of 80 CMD trajectories from TS2 and TS3 are included. (4.42 MB DOC) [file pcbi.1000998.s001.doc]

**Figure S1** Block analysis to show the convergence (within last 500.0 ns out of 1250.0 ns) of the REMD simulation. Total 1250.0 ns sampling of FSD-1 at 280 K are equally divided into 5 blocks for calculating the abundance of native structure (i.e. the Cα RMSD against NMR structure at 280 K is less than 3.0 Ǻ).

**FigureS2** Distribution of the four order parameters at 323K.

| **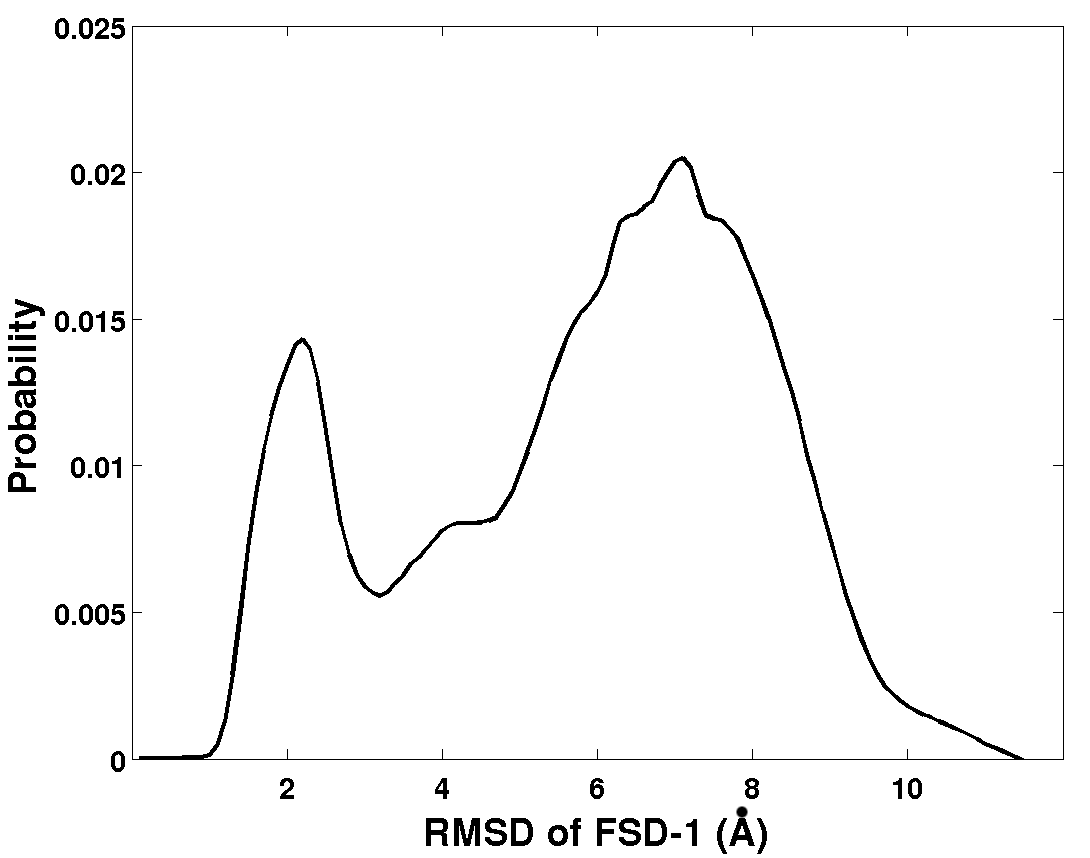** | **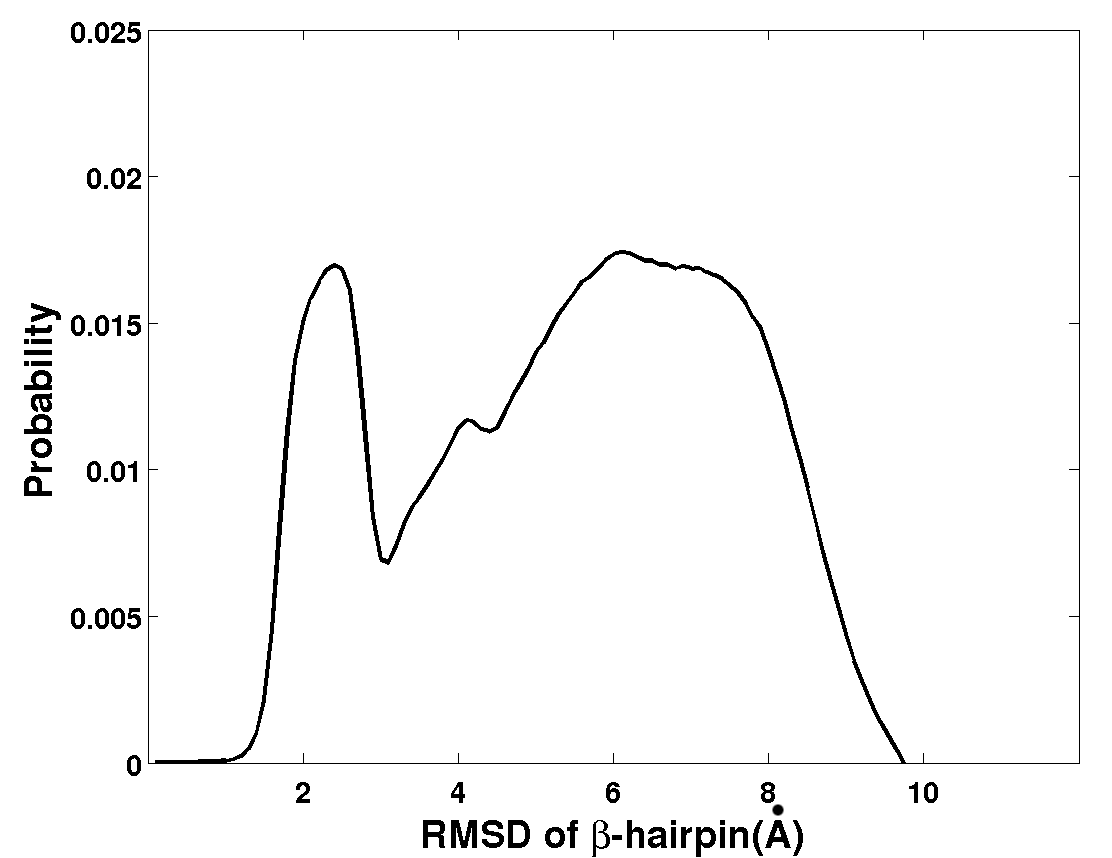** |
| --- | --- |
| **A** | **B** |
| **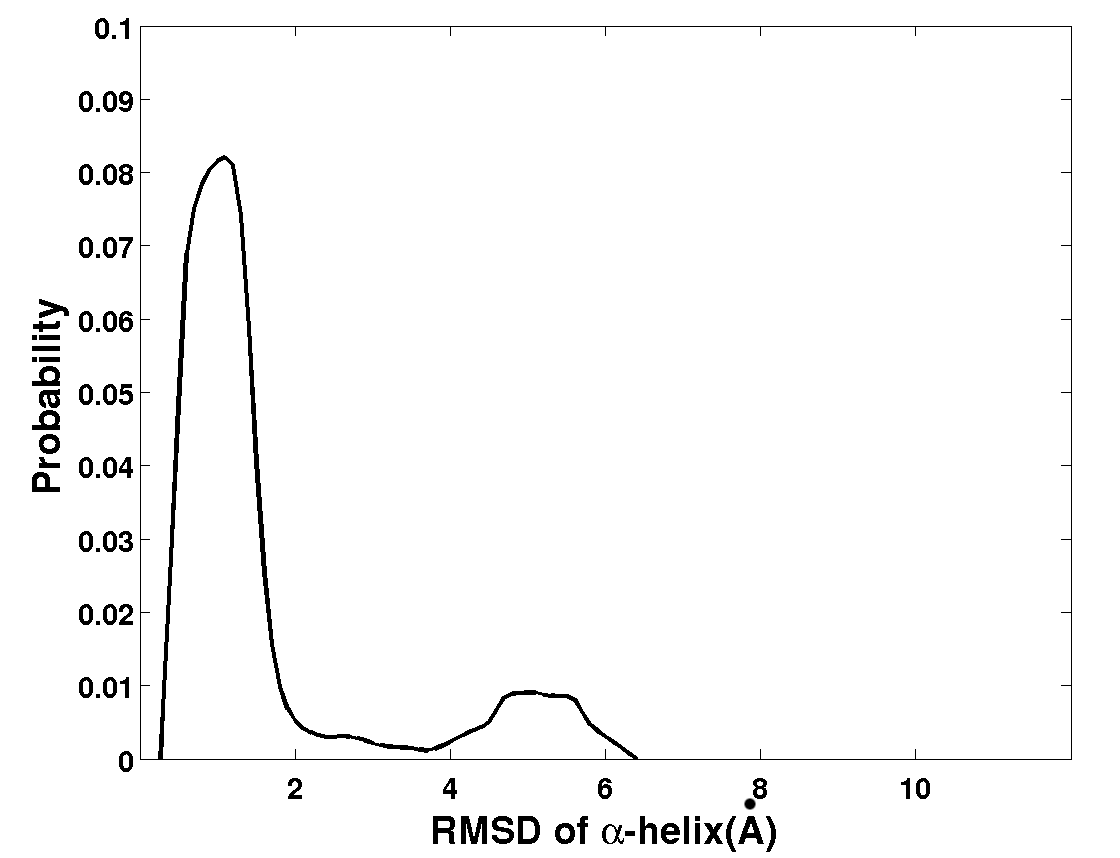** | **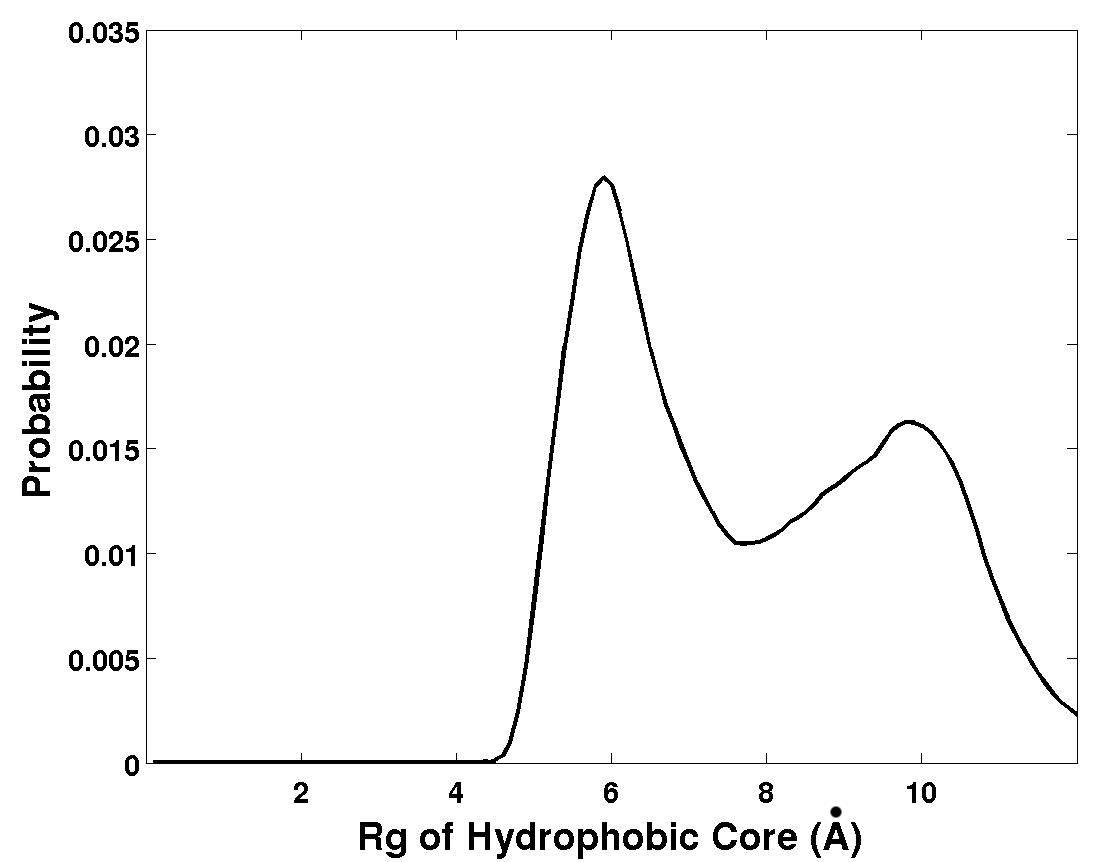** |
| **E** | **F** |

**FigureS3** The absolute heat capacity as a function of temperature from the REMD simulations using a Berendsen thermostat with a coupling constant of 2.0 ps.


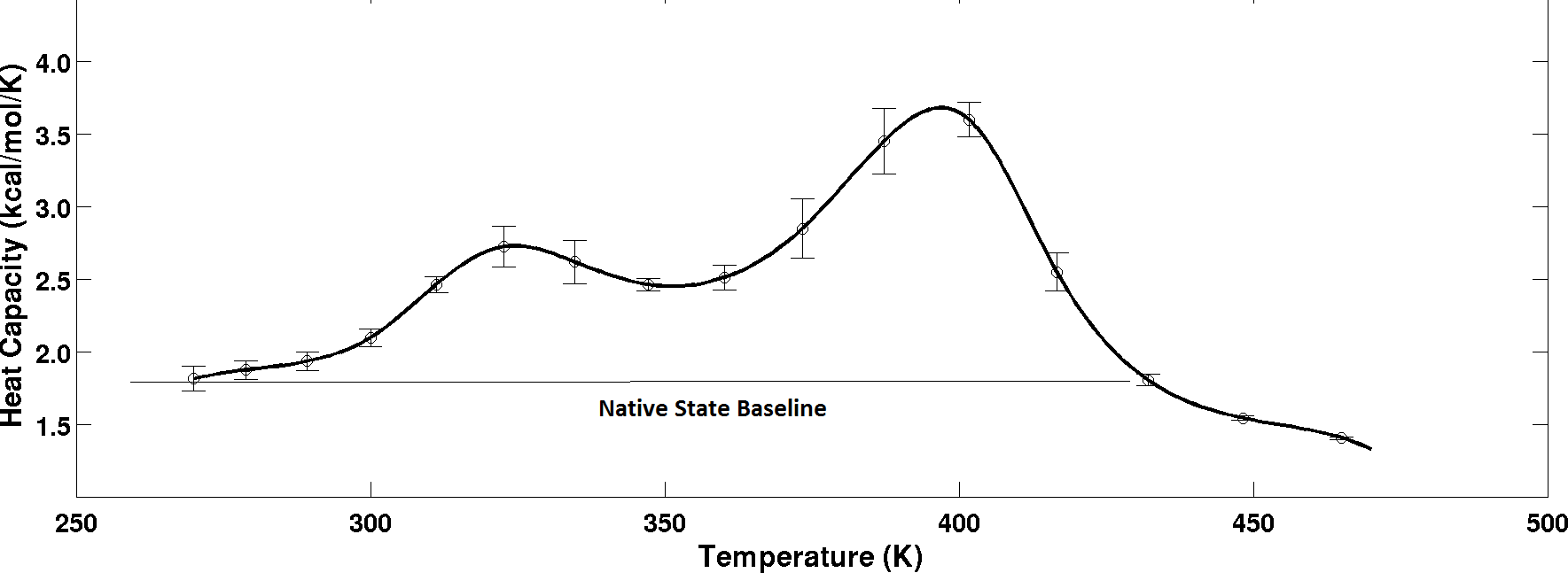


**FigureS4** Cα-RMSDs of 40 trajectories starting from the TS structure identified from the replica trajectory at 323K. A: trajectory 1-20 B: trajectories 21-40.


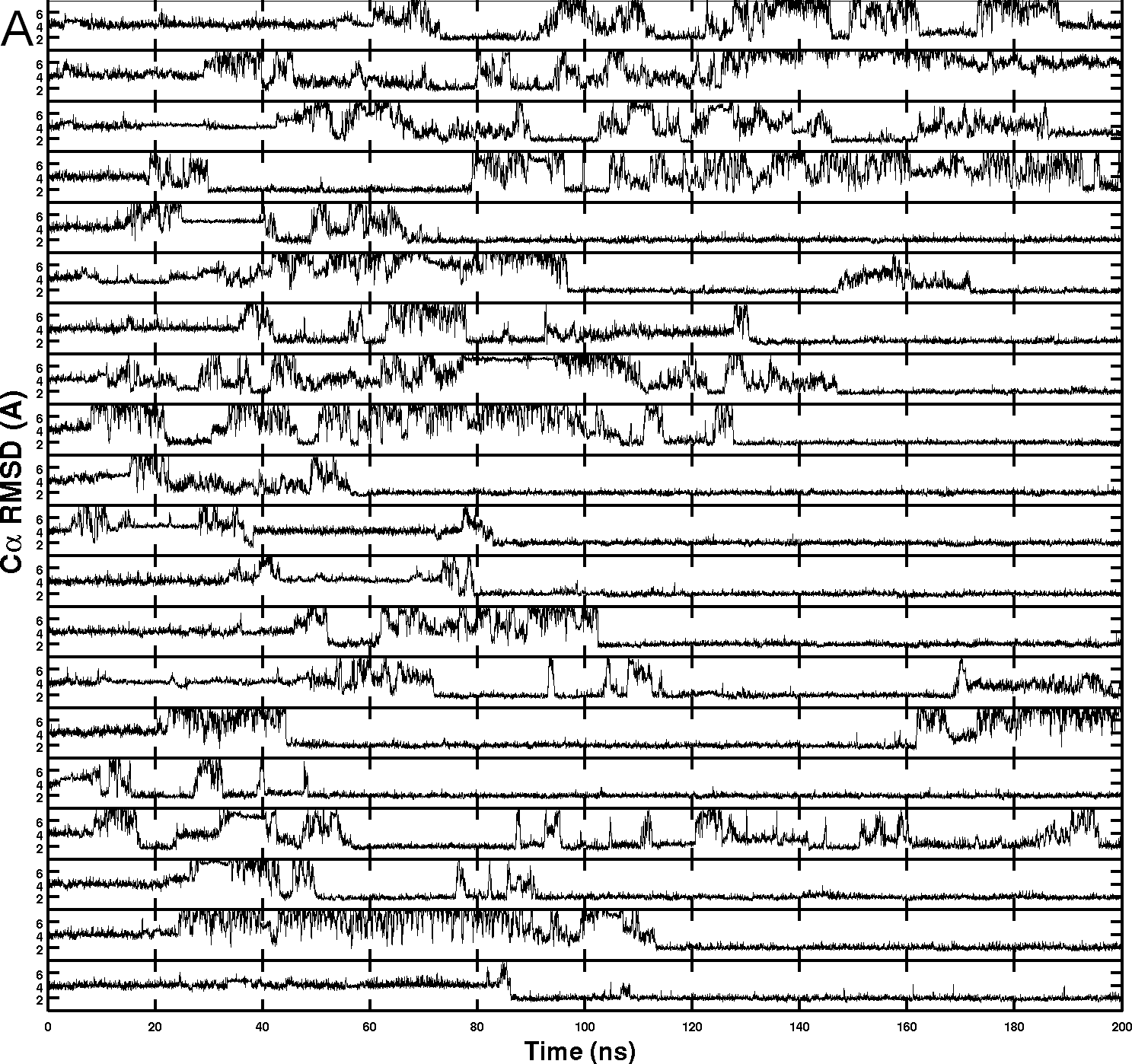


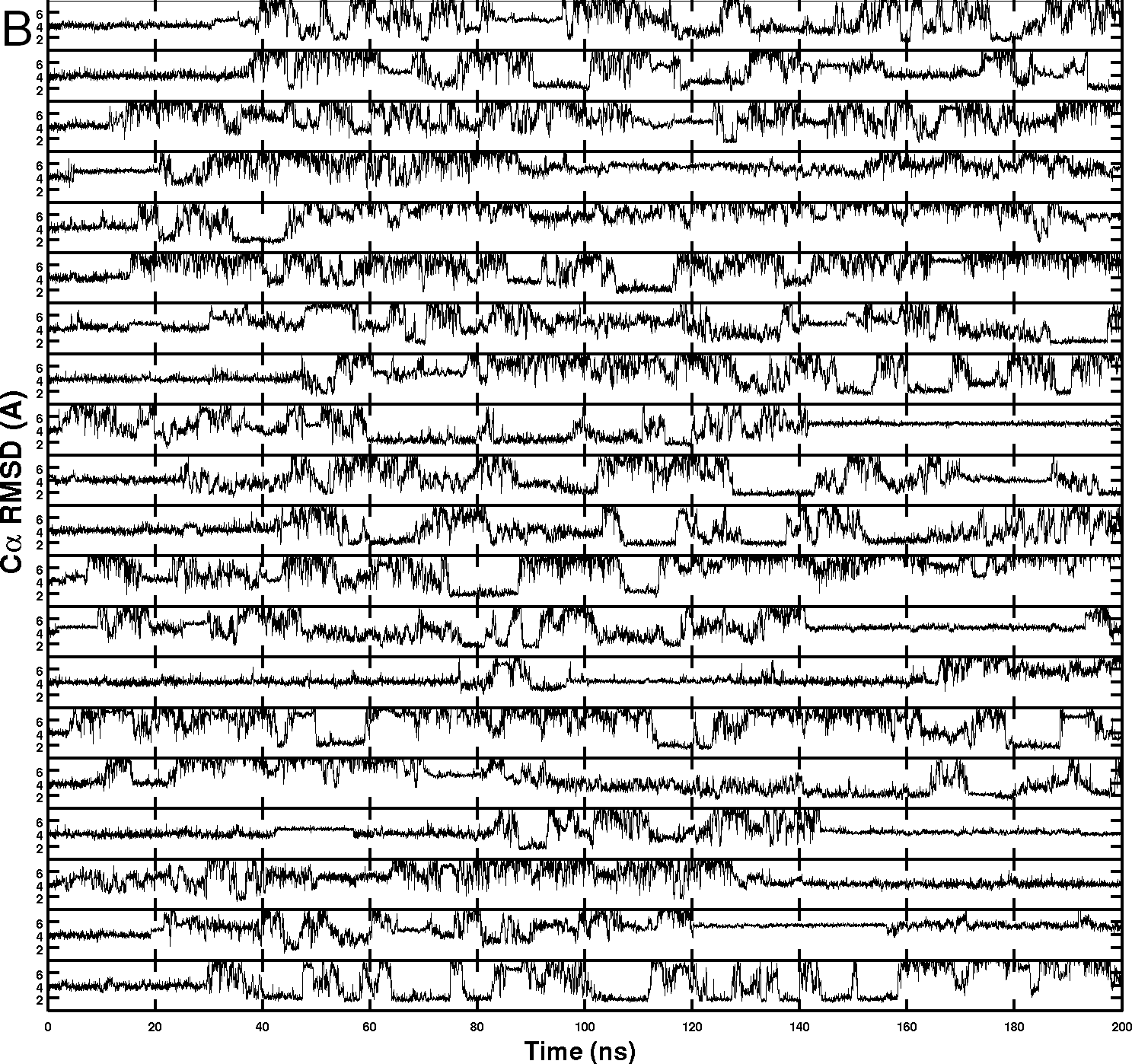


**Figure S5** The last snapshot of 20 CMD trajectories. The backbone is shown in cartoon and the secondary structure is coded by color: coil in silver, a-helix in purple, β-sheet in yellow, isolated β-bridge in tan and turn in cyan. The side chain type is also coded by color: hydrophobic in black, hydrophilic in green, positively charged in blue and negatively charged in red and N-terminal is shown by a red VDW ball.

| **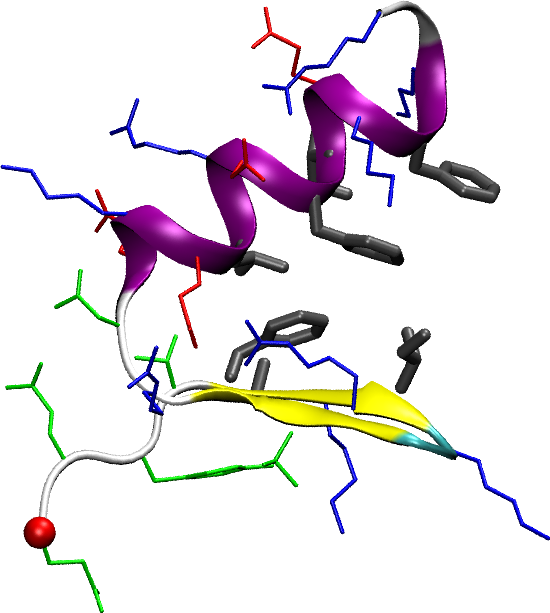** | **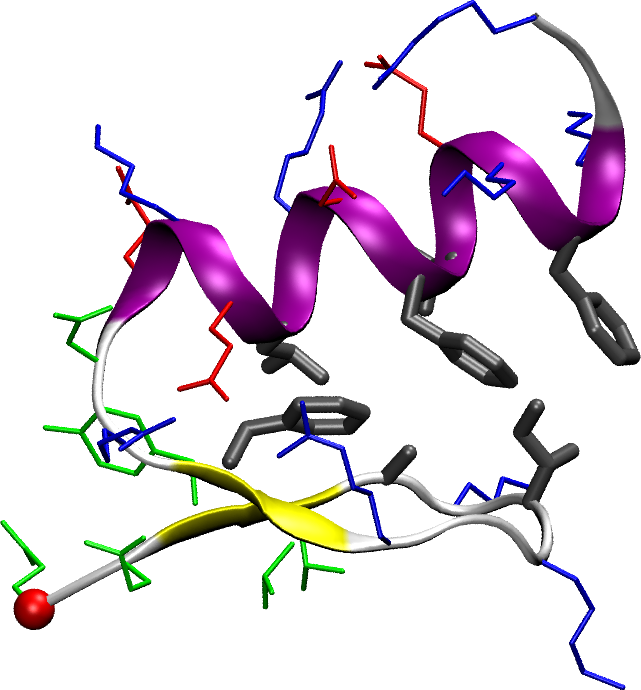** | **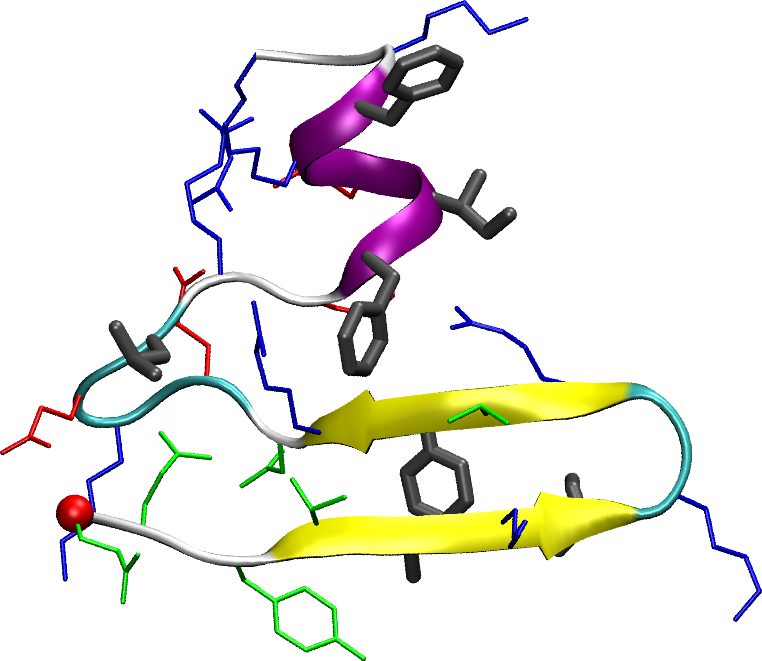** | **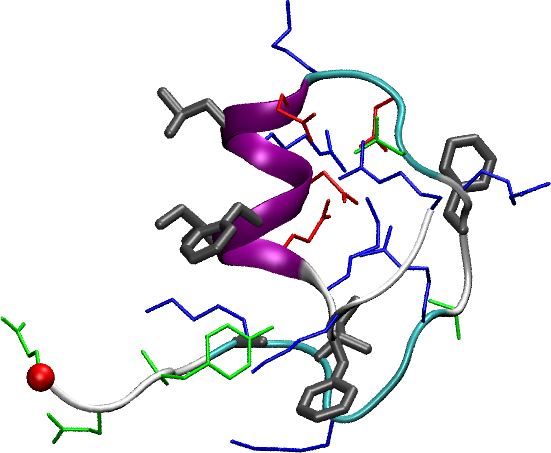** |
| --- | --- | --- | --- |
| **A** | **B** | **C** | **D** |
| **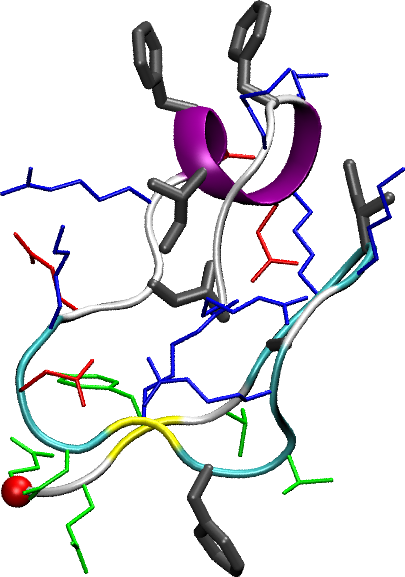** | **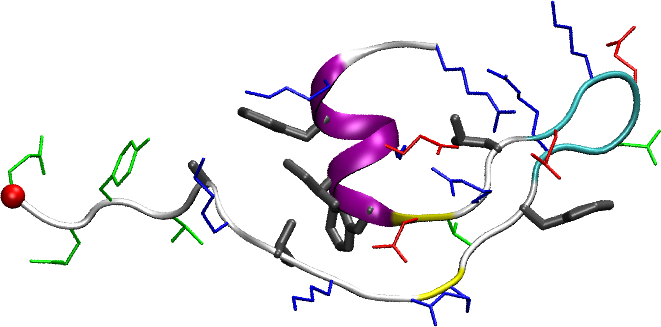** | **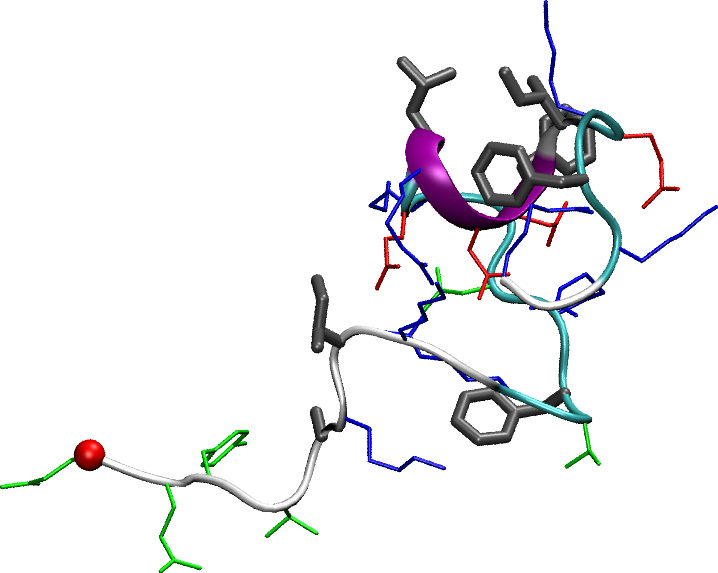** | **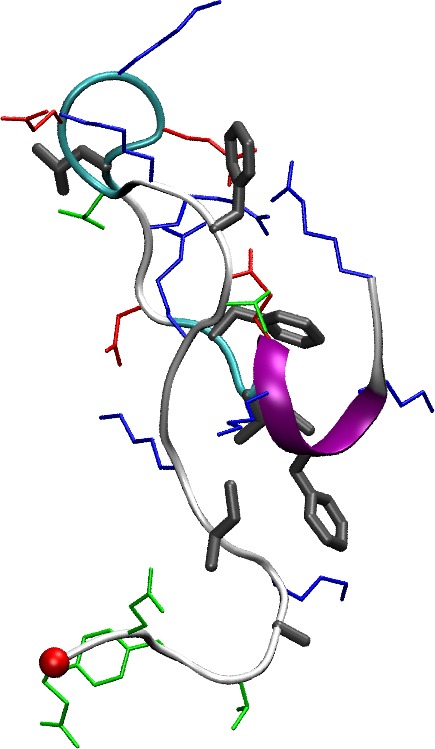** |
| **E** | **F** | **G** | **H** |
| **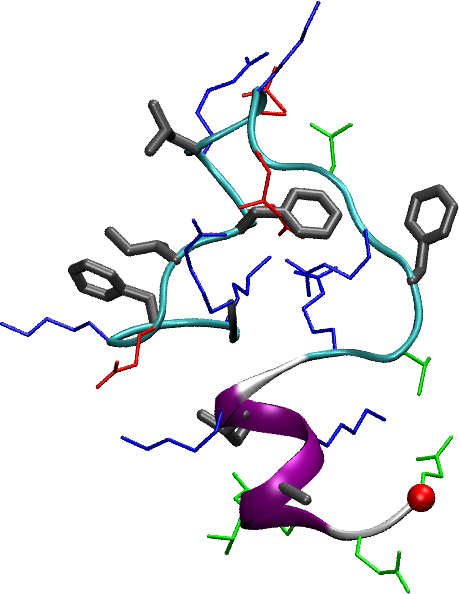** | **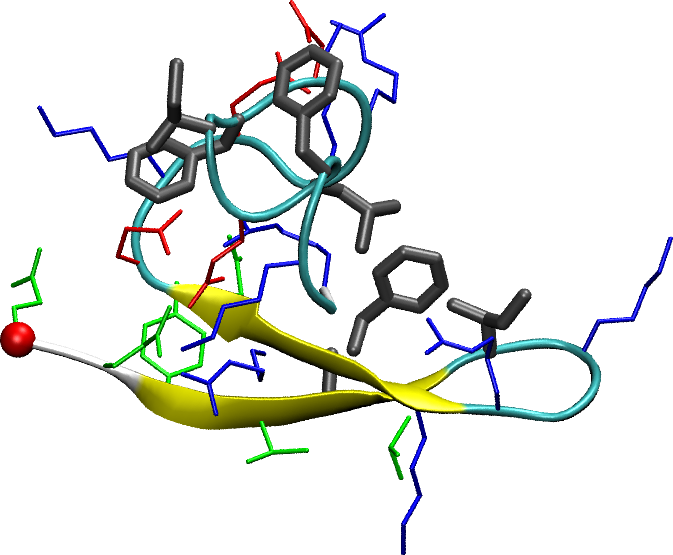** | **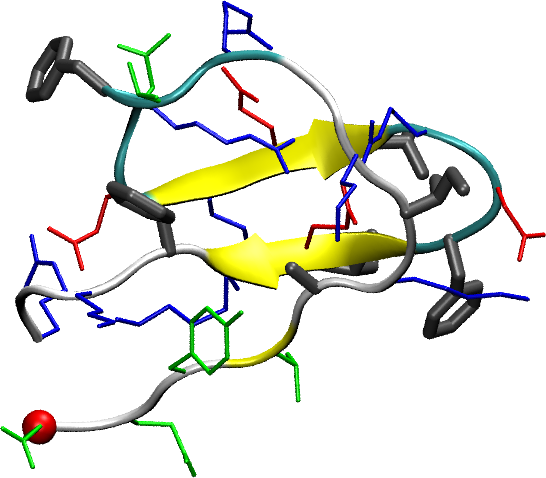** | **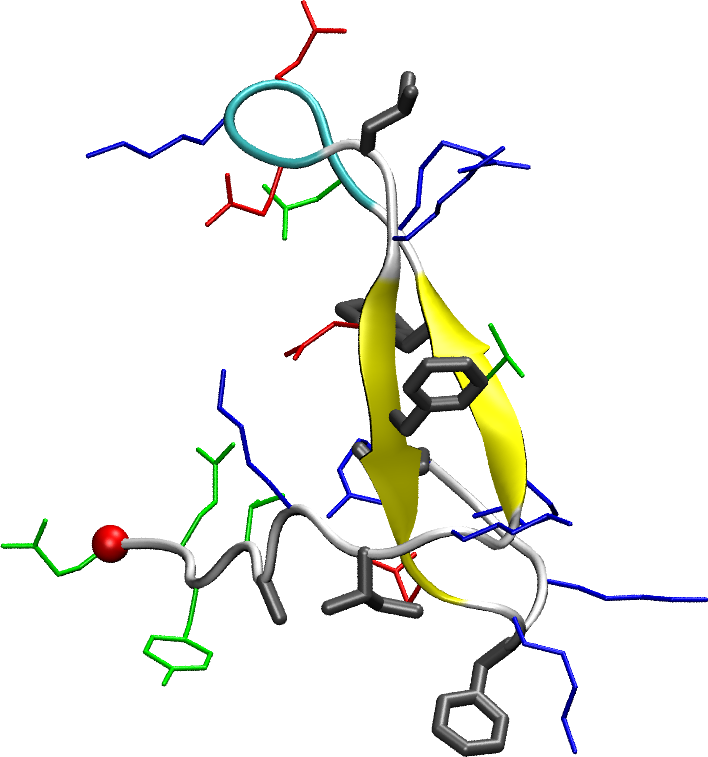** |
| **I** | **J** | **K** | **L** |
| **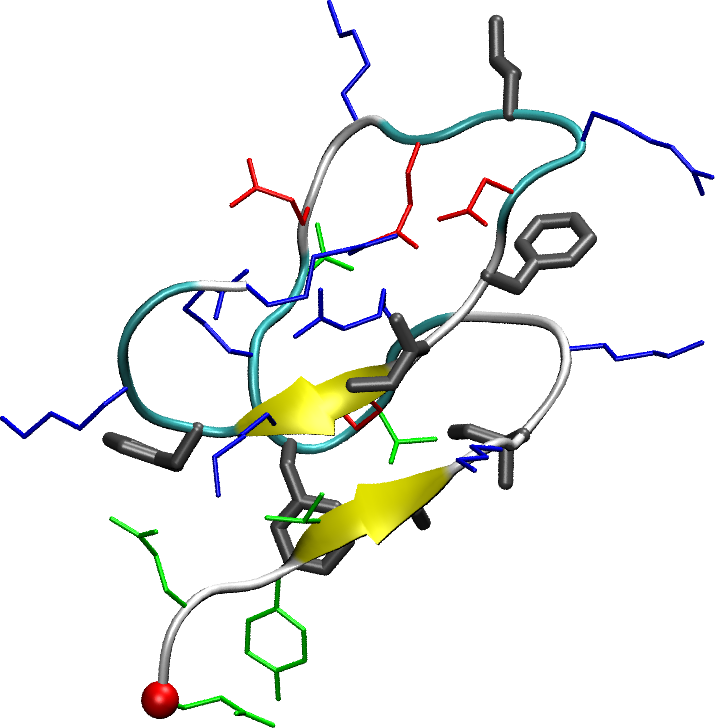** | **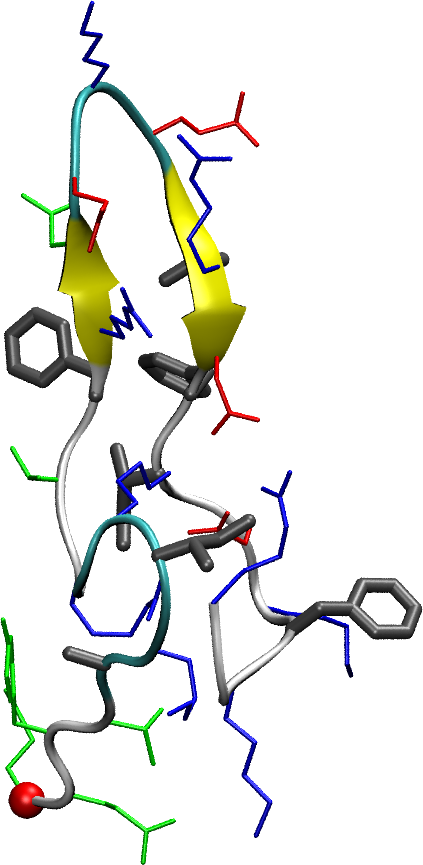** | **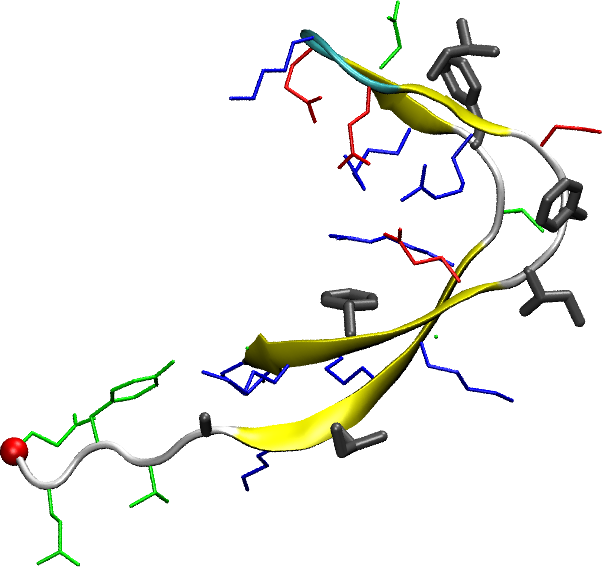** | **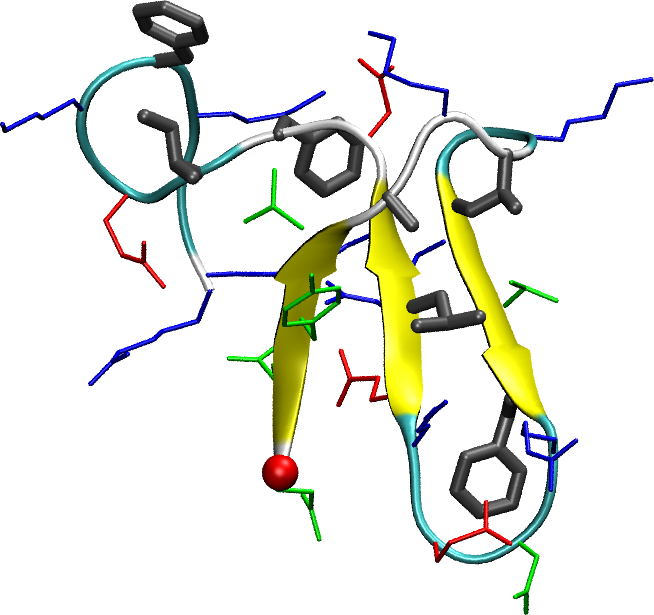** |
| **M** | **N** | **O** | **P** |
| **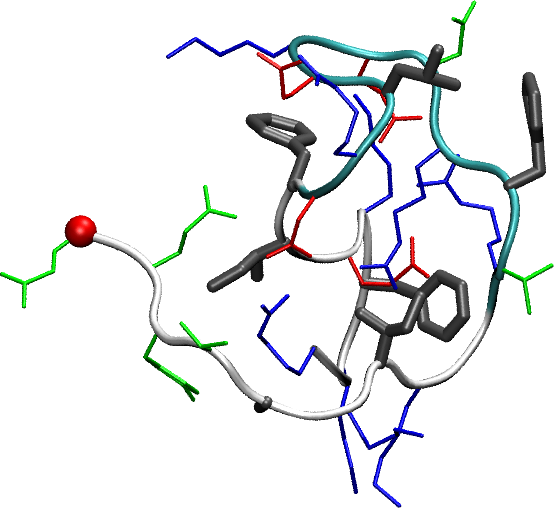** | **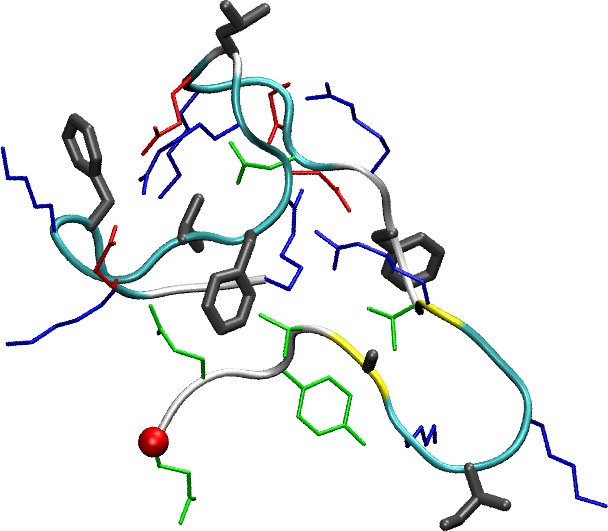** | **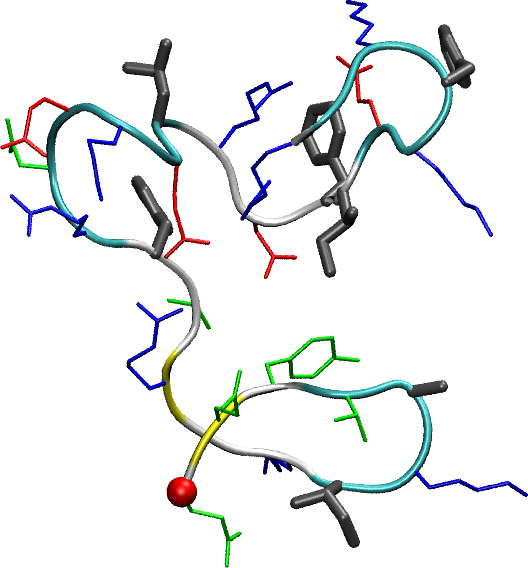** | **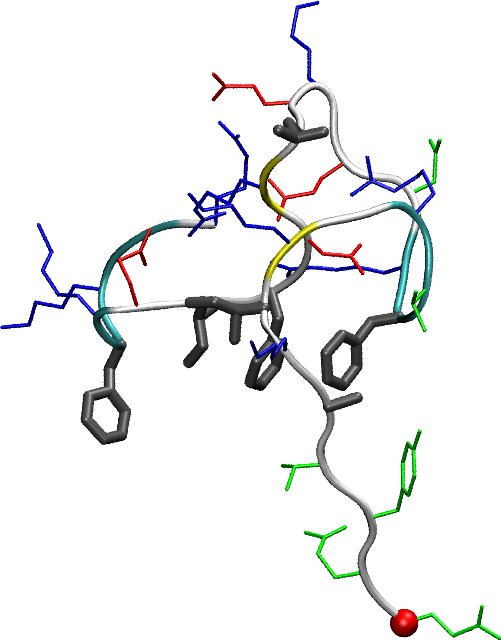** |
| **Q** | **R** | **S** | **T** |

**Figure S6** Cα-RMSDs of 20 CMD trajectories starting from an extended conformation at 300 K.

**
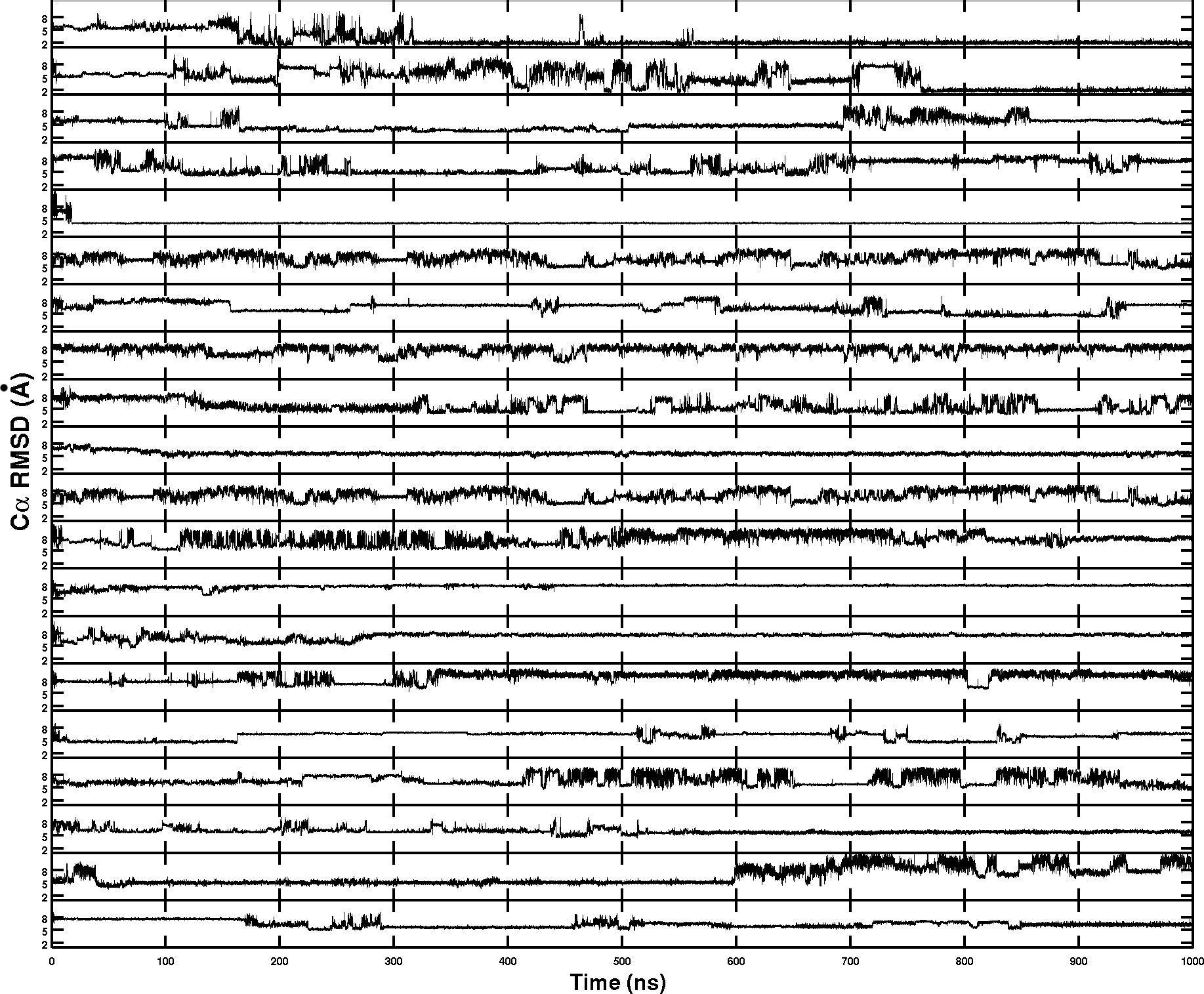
**

**Figure S7** Cα-RMSDs of 40 trajectories starting from the TS structure identified from the successful folding trajectory 1. A: trajectory 1-20 B: trajectories 21-40

**
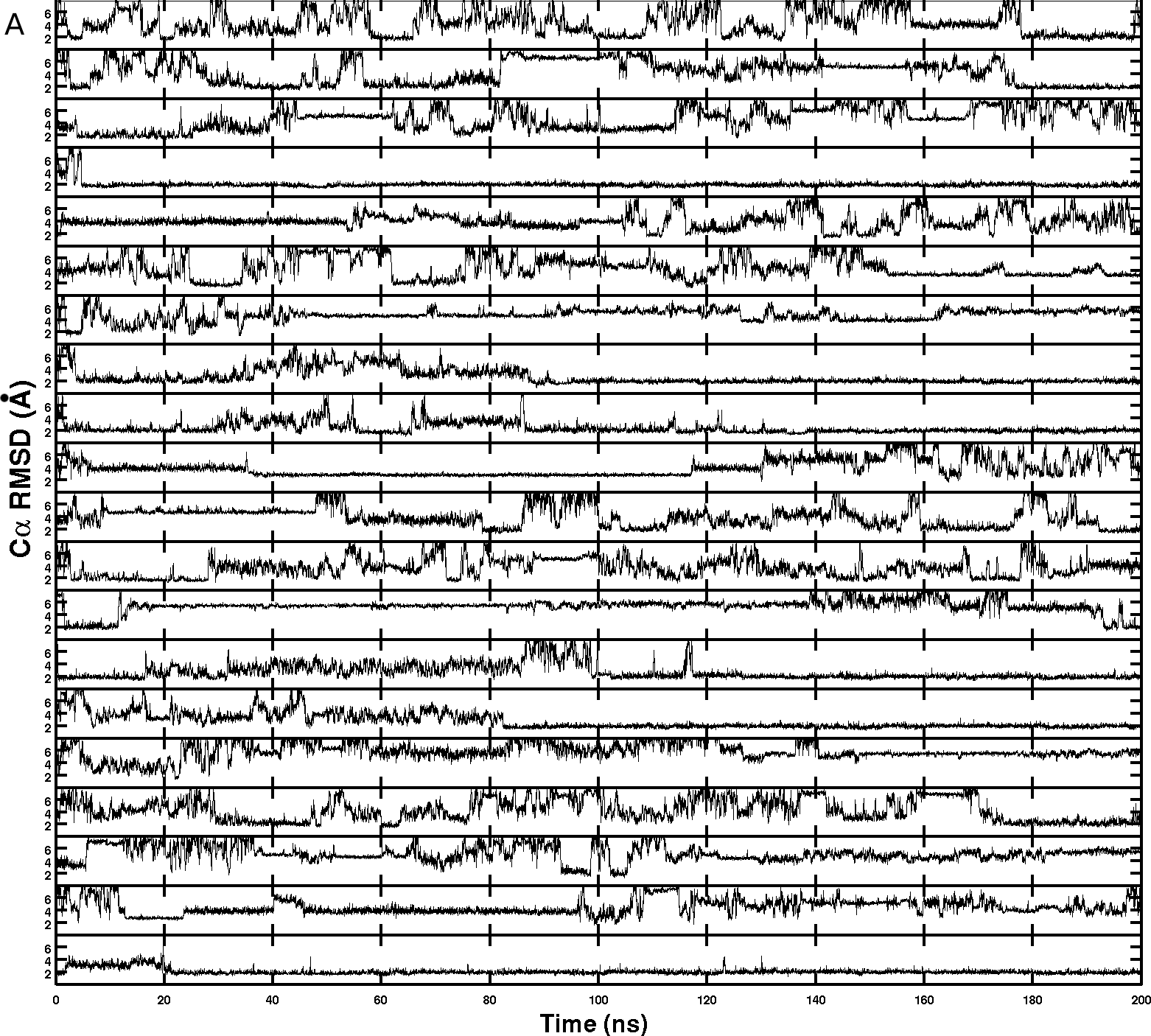
**

**
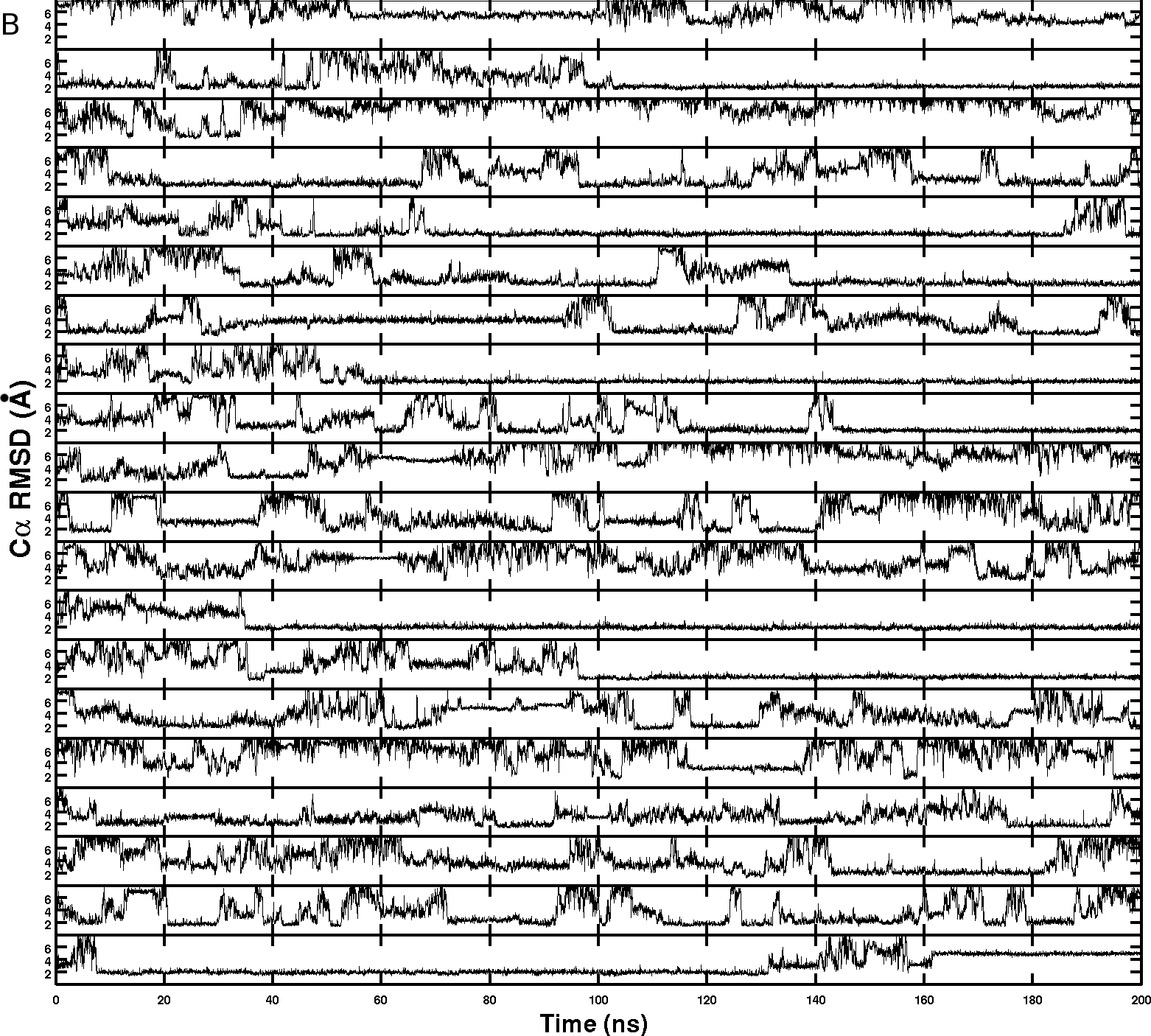
**

**Figure S8** Cα-RMSDs of 40 trajectories starting from the TS structure identified from the successful folding trajectory 2. A: trajectory 1-20 B: trajectories 21-40

**
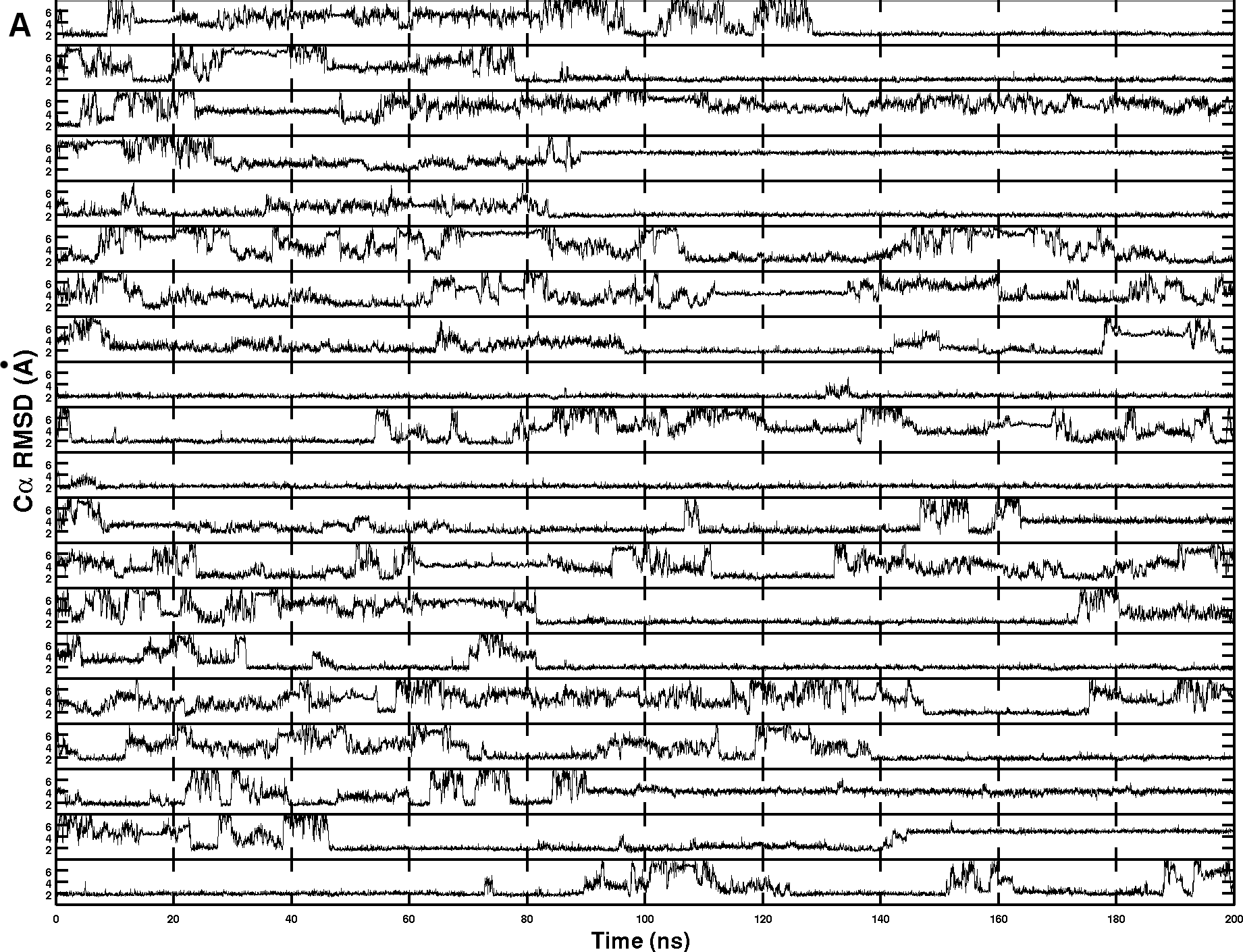
**

**
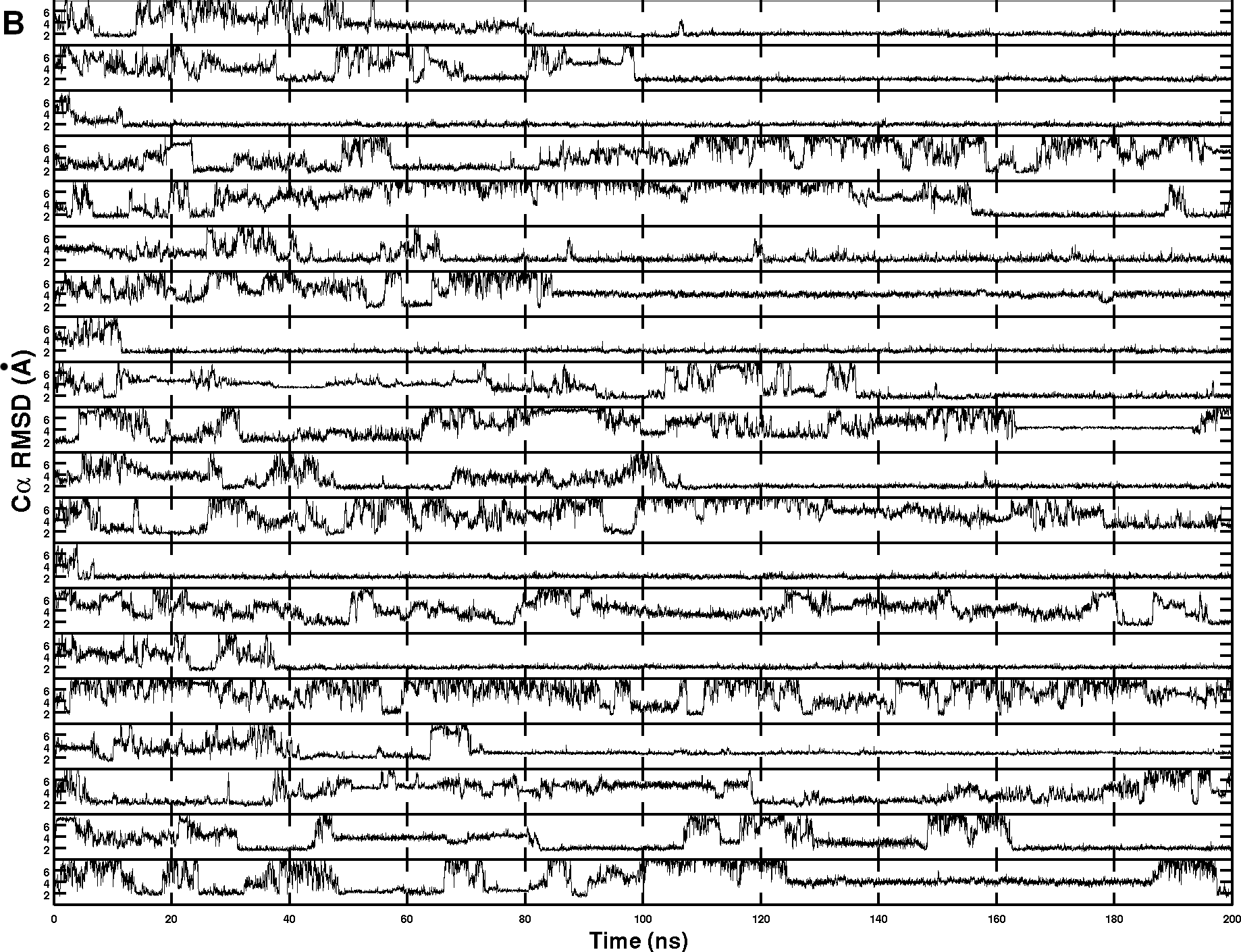
**
